# Molecular Simulations Matching Denaturation Experiments for N^6^-Methyladenosine

**DOI:** 10.1021/acscentsci.2c00565

**Published:** 2022-08-03

**Authors:** Valerio Piomponi, Thorben Fröhlking, Mattia Bernetti, Giovanni Bussi

**Affiliations:** Scuola Internazionale Superiore di Studi Avanzati, SISSA, via Bonomea 265, 34136 Trieste, Italy

## Abstract

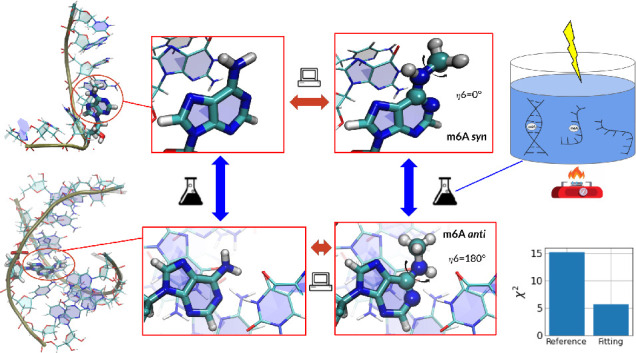

Post-transcriptional modifications are crucial for RNA function and can affect its structure and dynamics. Force-field-based classical molecular dynamics simulations are a fundamental tool to characterize biomolecular dynamics, and their application to RNA is flourishing. Here, we show that the set of force-field parameters for N^6^-methyladenosine (m^6^A) developed for the commonly used AMBER force field does not reproduce duplex denaturation experiments and, specifically, cannot be used to describe both paired and unpaired states. Then, we use reweighting techniques to derive new parameters matching available experimental data. The resulting force field can be used to properly describe paired and unpaired m^6^A in both *syn* and *anti* conformation, which thus opens the way to the use of molecular simulations to investigate the effects of N6 methylations on RNA structural dynamics.

## Abstract 2

We report a method to train force-field parameters on RNA melting experiments using alchemical simulations and derive parameters for m6A that can be used for both paired and nonpaired nucleotides.

## Introduction

Post-transcriptionally modified nucleotides are crucial for RNA function.^1,2^ Methylation of adenine in the N6 position (m^6^A) is the most prevalent chemical modification in mRNAs and has been observed in both coding and noncoding RNAs.^1−4^ m^6^A can finely regulate the interaction of RNA with specific proteins known as m^6^A readers. In addition, similarly to other chemical modifications,^5^ it can directly affect RNA stability and structural dynamics (see, e.g., refs (6−8)). Specifically, m^6^A has been suggested to weaken Watson–Crick pairings due to the incompatibility of its most stable conformation (*syn*) with duplex formation^9−11^ (see also Figure 1). Interestingly, recent nuclear magnetic resonance (NMR) experiments have identified *syn*/*anti* dynamics in both paired and unpaired m^6^A, recapitulating the effect of N6 methylation on RNA conformational kinetics.^11^ Molecular dynamics (MD) simulations give access to structural dynamics at the atomistic resolution^12^ and are thus an ideal tool to complement NMR studies. The capability of classical force fields to predict the dynamics of difficult structural motifs is steadily increasing.^12−14^ However, the number of applications of MD simulations to N6-methylated RNAs reported to date is still limited.^11,15−20^ Force fields parameters for m^6^A developed by Aduri et al.^21^ were determined in a bottom-up fashion and are compatible with the AMBER force field, which is widely adopted for RNA simulations.^12^ These parameters are the default choice for a structure refinement tool.^22^ However, they have been quantitatively validated against a limited set of experimental results.^18^ The availability of denaturation experiments on a number of m^6^A-containing duplexes^9,23,24^ calls for a more extensive validation of force-field parameters and, ideally, for fitting force-field parameters directly on experiments.^25^

**Figure 1:**
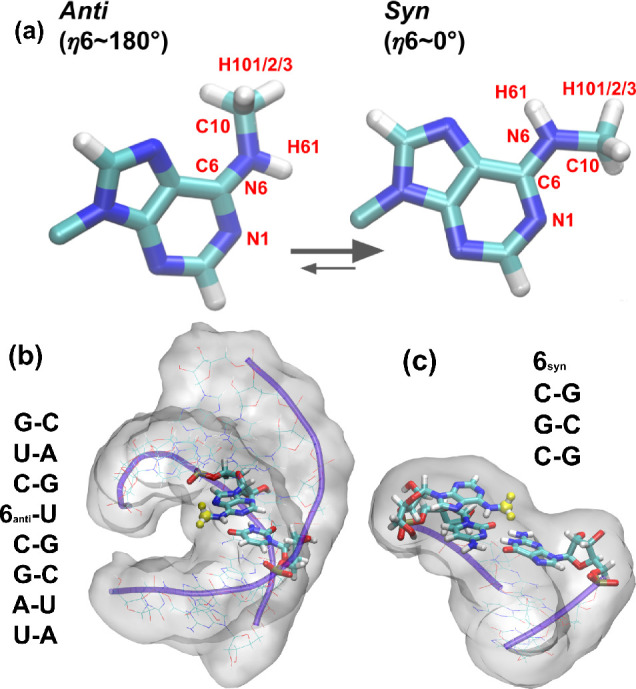
(a) N^6^-Methyladenosine (m^6^A) nucleobase in *anti* (less stable) and *syn* (more stable) conformations.^9,11^ Atom names in red correspond to charges reparametrized in this work. (b) Example of a duplex containing m^6^A in *anti* conformation, which is the expected conformation for the nucleotide when Watson–Crick paired. A 6 is used to denote m^6^A in secondary structures for compactness. (c) Example of a duplex with m^6^A as a dangling end in *syn* conformation. The m^6^A methyl group is highlighted in yellow.

In this paper, we validate and improve the parameters introduced in ref (21) by using alchemical free-energy calculations (AFECs).^26^ To this end, an unmodified adenine is converted to a modified one by switching *on/off* nonbonded interactions of specifically chosen atoms, in both single-stranded and double-stranded RNAs^18^ (see also Figure 2). We then develop a reweighting technique that can be used to predict results corresponding to a different set of charges without the need to perform new MD simulations. Additionally, we extend a recently introduced force-field fitting strategy^27^ to be usable in the context of alchemical simulations. The introduced approach allows training six charges and a dihedral potential so as to quantitatively reproduce methylation effects in denaturation experiments. The resulting force field can be used to properly describe paired and unpaired m^6^A in both *syn* and *anti* conformations.

**Figure 2:**
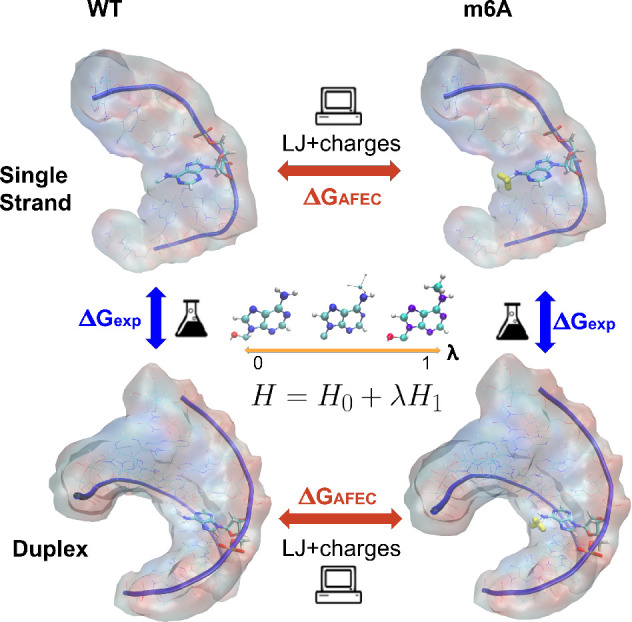
Thermodynamic cycle. Alchemical free-energy calculations (AFECs) allow computing *ΔG* by integrating along an alchemical path λ describing the transformation of nonmethylated adenosine into m^6^A, by switching *on/off* the nonbonded interaction of specifically chosen atoms. The relative free-energy change due to the modification can be estimated as the *ΔΔG* between AFECs performed on a duplex and on the corresponding single strand. This quantity can be directly compared to the difference in thermodynamic stability of duplexes with or without the modification, which can be measured experimentally through denaturation experiments.

## Material and Methods

### Simulated Systems

We simulated the isolated m^6^A nucleoside, 9 m^6^A-methylated duplexes for which denaturation experiments are available in the literature^9,24^ (see Table 1), and the corresponding single-stranded RNAs. For the isolated m^6^A nucleoside, we computed the *ΔG*_*syn*/*anti*_ by taking the difference in the *ΔG*s obtained with AFEC by methylating the adenosine in *syn* or *anti* conformations. We then chose several systems from ref (9). For systems A4 and A5, where m^6^A is present as a dangling end and thus unpaired, we only performed AFECs corresponding to the *syn* conformation. For the other systems, we performed AFECs in the expected *anti* conformation. For the A2 and A3 systems, we additionally performed AFECs in the unexpected *syn* conformation as a validation (population reported in ref (11) is ∼1%). In addition, we chose 5 more systems from ref (24), with the following criterion: they have a single methylation per strand, and the methylation occurs in an internal position of the duplexes. For all of these systems, we performed AFECs in the expected *anti* conformation.

**Table 1:** List of Systems Involved in the Fitting and Relative Experimental *ΔΔG*^a^

	system	*ΔΔG* (kJ/mol)
A1	m^6^A *ΔG*_*syn*/*anti*_	6.3^9^
A2	UACG6CUG	1.7 ± 0.9^9^
	AUGCUGAC	
A3	CGAU6GGU	7.1 ± 0.9^9^
	GCUAUCCA	
A4	6CGC	–2.5 ± 1.2^9^
	GCG	
A5	GCG6	–1.7 ± 0.9^9^
	CGC	
B1	GUC6CUG	2.5 ± 2.1^24^
	CAGUGAC	
B2	ACU6UAGU	2.1 ± 1.3^24^
	UGAU6UCA	
B3	AGUU6ACU	5.4 ± 1.3^24^
	UCA6UUGA	
B4	CGGUGC6UCG	8.6 ± 0.8^24^
	GCU6CGUGGC	
B5	ACUUA6GU	1.7 ± 1.0^24^
	UG6AUUCA	

a The first system is the single nucleotide, and the experimental value corresponds to *ΔG*_*syn*/*anti*_. In A2–A5 and B1–B5, the “6” in the double strand sequences is used to identify m^6^A for compactness. *ΔΔG*s for systems A2–A5 were measured by Roost et al.^9^ In A4 and A5, the m^6^A is positioned as a dangling end and has a stabilizing effect on the duplex. Experiments for systems B1–B5 were performed by Kierzek et al.^24^ In B2–B5 systems, the methylation occurs in both strands; however, the *ΔΔG*s reported are intended per methylation.

Starting structures for MD simulations were built using the proto-Nucleic Acid Builder.^28^ Single strands were generated by deleting one of the chains from duplex structures. All of the MD simulations were performed using a modified version of GROMACS 2020.3^29^ which also implements the stochastic cell rescaling barostat.^30^ The AMBER force field was used for RNA,^31−33^ the TIP3 model for water,^34^ and Joung and Cheatham parameters for ions.^35^ As a starting parametrization for m^6^A, we used AMBER adenosine parameters combined with modrna08^21^ charges for the nucleobase, adjusted to preserve the total charge of the nucleoside. Details on the implementation of these parameters and the initial tests are reported in Section S1. Charges are given in Table S2. We refer to this parametrization as the Aduri force field.

### Alchemical Simulations

For AFECs, we included a hybrid adenosine with double topology in the force-field definition: the first topology corresponding to standard adenosine, and the second one corresponding to m^6^A. We used 16 replicas in which Lennard-Jones (LJ) parameters and partial charges were simultaneously interpolated. In order to avoid singularities due to electrostatic interaction when the repulsive LJ potential is switched off,^26^ we used soft-core potentials as implemented in GROMACS (sc-alpha = 0.5, sc-sigma = 0.3, and sc-power = 1; see Section S2).^36^ Simulation boxes consist of rhombic dodecahedrons containing RNA, water, and Na^+^ and Cl^–^ ions with an excess salt concentration of 0.1 M. For a subset of the systems, further simulations were performed for a salt concentration of 1 M. The systems were energy-minimized and subjected to a multistep equilibration procedure for each replica: 100 ps of thermalization to 300 K in the NVT ensemble was conducted through the stochastic dynamics integrator (i.e., Langevin dynamics),^37^ and another 100 ps was run in the NPT ensemble simulations using the Parrinello–Rahman barostat.^38^ In production runs, the stochastic dynamics integrator was used in combination with the stochastic cell rescaling barostat^30^ to keep the pressure at 1 bar. Equations of motion were integrated with a time-step of 2 fs. Long-range electrostatic interactions were handled by particle-mesh Ewald.^39^ During production, a Hamiltonian replica exchange was used proposing exchanges every 200 fs. The set of λ values defining the replica’s Hamiltonians was chosen in such a way to guarantee transition probabilities above 20% and as homogeneous as possible (see Section S2), ensuring a sufficient phase space overlap between replicas. Each replica was simulated for 10 ns, for a total of 16 × 10 ns = 160 ns for each system. To decrease numerical errors in energy recalculations, trajectories were saved in an uncompressed format. At the end of the production phase, the 16 independent “demuxed” (i.e., continuous) trajectories were processed to recompute energies for each of the 16 Hamiltonian functions to compute *ΔG* via the binless weighted histogram analysis method (WHAM).^40−42^ Specifically, for each trajectory, a weight *w* was found for each snapshot *x* that allows computing statistics for the unmodified adenine as a weighted average over the set of concatenated replicas (see Section S3). These weights were then used to compute the *ΔG* associated with the methylation as
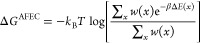
1where *ΔE*(*x*) = *E*_λ=1_(*x*) – *E*_λ=0_(*x*) is the difference between the total energy computed with the Hamiltonian energy functions associated with m^6^A and adenosine, respectively. We used a bootstrapping procedure to compute the statistical error on *ΔG* estimates by resampling the 16 continuous trajectories 200 times with replacement.^43^ As a control, we computed *ΔG*s using the standard Bennett-acceptance-rate estimate implemented in GROMACS.^44,45^ As can be seen in Table S3, these estimates are numerically equivalent to those obtained with binless WHAM.

*ΔΔG*s were obtained taking the difference between *ΔG*s obtained by methylating the adenosine in *anti* or *syn* conformation on the duplex or dangling end, respectively, and the *ΔG* obtained methylating in *syn* conformation on the relative single strand. Transitions between *syn* and *anti* states were never detected during the alchemical simulations. In this way, the contribution to the free energy given by the *syn* (*anti*) conformation in the duplex (single strand or dangling end) was ignored. Indeed, we expect these contributions to be negligible based on the experimental evidence,^9,11^ which show a *syn/anti* isomer preference when paired (∼1:100) versus unpaired (∼10:1). This was additionally verified with supplementary simulations performed on the A2 and A3 systems (see Table S3). Moreover, we computed *ΔG*_*syn*/*anti*_ by performing the alchemical transformations on the isolated nucleoside in solution for the two isomers and computing their difference (see Table S3).

### Fitting Procedure

We employ a fitting strategy based on reweighting^27^ where a subset of the partial charges and a dihedral potential are adjusted to match experimental data. Specifically, we decided to fit the atoms that are closer to the methyl group (N6, C6, H61, C10, H101/2/3, and N1, see Figure 1). The total charge was maintained, leading to 5 free parameters associated with the partial charges. A single cosine was added to the η_6_ torsional angle identified by atoms N1–C6–N6–C10: *U*(*x*) = *V*_η_ [1 + cos(η_6_(*x*) – π)]. This angle controls the *syn/anti* relative populations, leading to a total of 6 parameters, and the shift is chosen so that a positive value of *V*_η_ favors *syn* configurations over *anti*.

To optimize the calculation of the total energy of the system at every iteration of our fitting procedure, where up to 6 charges were possibly modified, we notice that the total energy of the system is a quadratic function of the charge perturbations *ΔQ*_*i*_. Without loss of generality, one can write the energy change associated with charges and torsion perturbation as
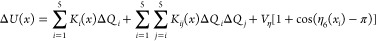
2In total, for every analyzed snapshot (*x*), 20 coefficients (*K*_*i*_ and *K*_*ij*_) can be precomputed that allow obtaining the energy change for arbitrary choices of *ΔQ* with simple linear algebra operations, without the need to recompute electrostatic interactions explicitly. The coefficients were obtained by using GROMACS in rerun mode for 20 sets of test charge perturbation, which were extracted from a Gaussian with zero average and standard deviation set to 1 *e*. The perturbations were constructed to maintain constant the total charge. Importantly, this approach correctly takes into account the effect of charge perturbations on 1–4 interactions, where electrostatics is scaled with a force-field-dependent fudge factor, as well as on 1–2 and 1–3 interactions, for which it is discarded, and interaction with all the periodic images. The second order expansion above is exact if one neglects round-off errors. The magnitude of charge perturbations was chosen to minimize such errors. Equation 1 should then be suitably modified replacing *ΔE* with *ΔE* + *ΔU*. Its derivatives with respect to the free parameters (charge and dihedral potential coefficient) can be computed as well (see Section S6).

Our fitting is based on the minimization of an L2-regularized cost function defined as follows:
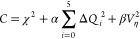
3
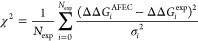
4Here, σ_*i*_ corresponds to the experimental error, and the χ^2^ measures the discrepancy between computations and experiments, whereas the regularization terms on the charges and the torsional η_6_ are governed by the hyperparameters α and β and are needed to avoid overfitting on the training set. This function is minimized using the L-BFGS-B method^46^ as implemented in SciPy.^47^

The result crucially depends on the choice of the hyperparameters α and β. Lower values for the hyperparameters imply that larger corrections are allowed, with the risk of overfitting, and thus lower transferability to new experiments. Higher values for the hyperparameters imply that lower corrections are allowed, with the risk of underfitting and thus lower accuracy in reproducing experimental data. The sweet point could be in principle found with a cross-validation (CV) procedure and a scan over possible values for α and β.^25,27^ For the smallest data set (set A in Table 1), we used a leave-one-out CV strategy; i.e., we trained the parameters on all systems except one. For the largest data set (set AB in Table 1), we used a leave-3-out strategy, iteratively training the parameters on 7 randomly chosen experiments and validating on the 3 left-out experiments. In both cases, we then assessed the transferability of the model by evaluating its average χ^2^ on the system (or the subset of systems) that was left out.

### Statistical Significance

When recomputing energies through a reweighting procedure, particular attention must be taken toward the statistical significance that may be lost during the computation, by reducing the effective sample size of the data set. This is usually monitored by computing the Kish effective sample size.^48,49^ In our case, the most affected ensemble is the one corresponding to m^6^A (λ = 1). We thus monitor the Kish size computed using weights corresponding to the λ = 1 ensemble, defined as
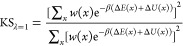
5We then compare it with the Kish size obtained with the original force field, defined as
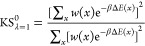
6To quantify how much statistical efficiency is lost due to the reweighting to a modified set of parameters, we use the Kish size ratio (KSR) that we define as
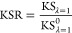
7

## Results

In this work, we fitted point charges and a single torsional potential correction for an m^6^A RNA residue using alchemical MD simulations and a set of experimental data, following the scheme shown in Figure 2. In all of the fittings, charges and torsional potential were subject to L2 regularization with hyperparameters α and β, respectively. We initially employed only the first 5 experimental data points of Table 1, namely, (A1) *ΔG*_*syn*/*anti*_ for a nucleobase and (A2–A5) *ΔΔG* in melting experiments.^9^ Thus, we first report the results obtained with such a set of charges, including a validation done on a more recent set of melting experiments (B1–B5).^24^ We then report results obtained with charges that were fitted on the entire data set (A1–A5 and B1–B5). As a reference, results obtained with the Aduri et al.^21^ modifications (modrna08) for the commonly used AMBER force field are also reported, either *as is* or complemented with a custom torsional correction that results in a *ΔG*_*syn*/*anti*_ matching experiment A1. All of the calculated *ΔG*s are reported in Table S3. A complete list of the performed alchemical simulations is reported in Section S9. Structural snapshots for all simulation systems are reported in Figures S9–S18. We note that, in a preliminary version of this work, some of the results were obtained by reweighting simulations performed with a slightly different set of charges, obtained by an incorrectly regularized fitting. The original results are very close to those reported here and can be seen in the preprint available at https://arxiv.org/abs/2203.14886v2.

### Fitting on the Smaller Data Set

For this first fitting, we only employed data set A1–A5 from Table 1. χ^2^ errors were computed using eq 4 and setting the experimental error of each data point (σ_*i*_) to be equal to each other and to 1 kJ/mol.

Figure 3a reports the results of a cross-validation test performed with a leave-one-out procedure. Namely, we fit the whole experimental data set leaving out one experimental data point at a time and report the average error on the left-out experiment. In this leave-one-out procedure, we decided not to iterate on the *ΔG*_*syn*/*anti*_ experiment (A1), since this is expected to be crucial to correctly reproduce the conformation of non-Watson–Crick-paired residues (mostly *syn*). From this map, we can hardly appreciate any variation of the χ^2^ along the vertical axis corresponding to the β hyperparameter. This suggests that β could be set to zero, thus simplifying all subsequent hyperparameter scans. Conversely, the χ^2^ grows significantly for low α values. This implies that regularization of charges is required to avoid overfitting. In general, one should expect a minimum to be observed in this type of hyperparameter scan.^25,27^ This is not the case here for the α scan (see also Figure S3, showing projection on α for β = 0), implying that the performance of the parameters on a given system is not improved when excluding that system from the training set. This is likely due to the small data set employed.

**Figure 3:**
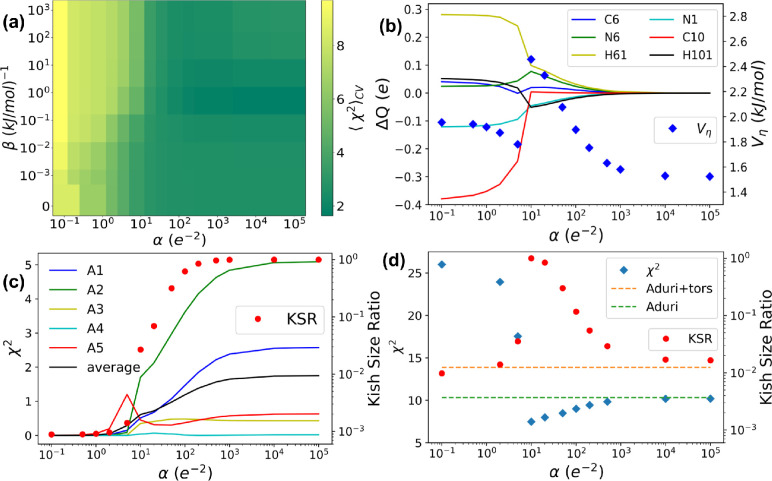
Results obtained with parameters fitted on the initial data set, A1–A5 in Table 1. (a) Cross validation error obtained with a leave-one-out-procedure, shown as a function of the two regularization hyperparameters α, for charges, and β, for the torsional potential. Darker green colors correspond to lower values of the average χ^2^ computed on the systems left out iteratively from the fitting. (b) Parameters (*ΔQ* and *V*_η_) obtained from the entire initial data set as a function of α, with β = 0. (c) χ^2^ errors for individual experiments and Kish size ratio (KSR, see text for definition) obtained using parameters fitted on the entire initial data set as a function of α, with β = 0. (d) Validation on the second data set (B1–B5 in Table 1) of the parameters obtained on the first data set. Results using Aduri parameters are shown as horizontal lines, either as reported in the original paper (green) or including a single torsional correction to obtain the correct *syn/anti* population (data point A1).

Figure 3b shows the optimized parameters (charge and torsional corrections) as a function of the regularization hyperparameter α while fixing β = 0. A transition can be seen at α ≈ 10. Namely, when α > 10, parameters have a smooth dependence on α, whereas when α < 10, both the charges and the torsional potential change suddenly. In the limit α → ∞, it can be seen that charge corrections tend to zero with an inverse law dependence, which is expected for L2 regularization, and the torsional correction tends to *V*_η_ ≈ 1.5 kJ/mol, which corresponds to the amplitude of the torsional potential that optimizes the χ^2^ without modifying the charges of the reference Aduri et al. model. We notice that *ΔG*_*syn*/*anti*_ obtained when using the Aduri et al. force field is ∼1.7 kJ/mol, and thus, this correction results in *ΔG*_*syn*/*anti*_ ≈ 1.7 + 2 × 1.5 = 4.7 kJ/mol, which is still smaller than the experimental reference ∼6 kJ/mol. The obtained parameters indeed strike a balance between favoring the *syn* state in the isolated nucleoside and not favoring it too much in the single-stranded calculations used to predict the *ΔΔG* from melting experiments, which would lead to too large destabilizations associated with the methylation. When α is decreased, the optimal torsional correction changes, since all of the parameters are coupled. This confirms that charges and torsional parameters should be fitted simultaneously.

Figure 3c shows the individual χ^2^ associated with the same hyperparameter scan. The average χ^2^ error is, by construction, monotonically increasing with α, and most of the individual errors follow the same trend. Figure 3c also shows the statistical efficiency of the analysis, quantified by the relative reduction of the Kish effective sample size associated with reweighting. A low number here indicates that the tested charges are so different from those employed in the simulation to make the result statistically not significant. The Kish size displays a significant drop for α < 10, indicating that results in this regime might be not significant. This is a likely explanation for the discontinuous behavior observed in Figure 3b.

We then tested the charges obtained with this reduced training set on the newer data set B1–B5 (see Table 1), which was not included in the training phase. This set of data involves 5 recently published melting experiments,^24^ 4 of which have m^6^A occurring in both chains of the duplex. We notice that double methylations are expected to lead to an even lower statistical efficiency of the reweighting procedure. We thus performed this analysis by reweighting simulations that were generated using the set of parameters derived fitting on systems A1–A5 for α = 10 and β = 0. Since this parametrization is closer to the right solution of the fitting when compared with the Aduri one, it obtains higher Kish size values in the relevant α range (see Figure 3d). The χ^2^ computed on the second data set shows that an optimal result can be obtained by setting α ≈ 10. We also compared with results obtained using the original Aduri charges and optionally including a torsional correction to fix the *syn/anti* balance. These results are obtained with direct simulation, that is, without reweighting. It can be seen that the results with the parameters trained on systems A1–A5 largely outperform those obtained with Aduri parameters on systems B1–B5, thus confirming the transferability of the parameters. Aduri+tors parametrization corresponds to setting *V*_η_ = 2.35 kJ/mol in such a way to perfectly fit experiment A1 (single nucleoside) without modifying charges. The χ^2^ computed for Aduri+tors demonstrates that acting exclusively on the torsional is not sufficient to reproduce both *ΔG*_*syn*/*anti*_ and melting experiments. It is also important to note that the improvement in reproducing experiments is obtained by changes in the partial charges that are small when compared to differences between charges derived with the standard restrained electrostatic potential protocol^50^ in different conformations (see Section S5).

### Fitting on the Full Data Set

Next, we perform a fitting using the full data set reported in Table 1. Since the variability of error in this data set is larger, we here computed χ^2^ using the experimental errors reported in Table 1. For the *ΔG*_*syn*/*anti*_ experiment, for which an experimental error is not reported, we used a nominal σ = 0.5 kJ/mol to assign to this experiment a larger weight when compared to the other data points corresponding to melting experiments.

Figure 4a reports the results of a cross-validation test performed with a leave-three-out procedure. Namely, we randomly select seven systems to be used in training, and we report the average χ^2^ error obtained for the remaining three systems. This time, system A1 was also allowed to be left out of the training set. Results are qualitatively consistent with those obtained with the smaller data set (see Figure 3). It is difficult to appreciate any variation of the χ^2^ along the vertical axis corresponding to the β hyperparameter, suggesting that we can safely set β = 0. We also do not find any clear minimum when scanning over α (see also Figure S4, showing projection on α for β = 0). Figure 4b shows the parameters as a function of the regularization hyperparameter α while fixing β. A clear transition can be seen at α ≈ 20. The average χ^2^ error is monotonically increasing with α, but some of the systems have a nontrivial behavior (Figure 4c). The Kish size shows a significant drop for α < 50, showing that results in this regime might not be statistically reliable. We thus select the parameters obtained with α = 50 as the optimal ones trained on the entire data set.

**Figure 4:**
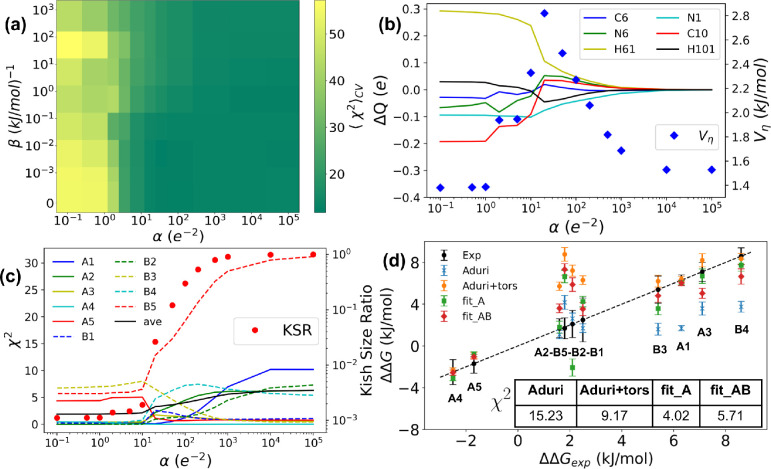
Results obtained with parameters fitted on the full data set, A1–A5 and B1–B5 in Table 1. (a) Cross-validation error obtained with a leave-three-out-procedure, shown as a function of the two regularization hyperparameters α, for charges, and β, for the torsional potential. Darker green colors correspond to lower values of the average χ^2^ computed on the systems left out iteratively from the fitting. (b) Parameters (*ΔQ* and *V*_η_) obtained from the entire data set as a function of α, with β = 0. (c) χ^2^ errors for individual experiments and Kish size ratio (KSR, see text for definition) using parameters fitted on the entire initial data set as a function of α, with β = 0. (d) *ΔΔG* computed for each of the 10 analyzed systems with 4 different sets of parameters. fit_A is parameters obtained fitting on the first data set (A1–A5) with regularization α = 10. fit_AB is derived fitting on the entire data set (A1–A5 and B1–B5) for α = 50. χ^2^ values obtained for each force field set of parameters are shown in the table inside panel d.

We then compare the performance of several different sets of parameters in reproducing all of the available experimental data points. Namely, we compare (a) the original Aduri parameters (Aduri), (b) the Aduri parameters augmented with a torsional correction to enforce the correct *syn/anti* balance in a nucleobase (Aduri+tors), (c) the parameters obtained fitting on the initial data set (A1–A5) with hyperparameter α = 10 (fit_A), and (d) the parameters obtained fitting on the full data set (A1–A5 and B1–B5) with hyperparameter α = 50 (fit_AB). Free energies are computed directly from the alchemical simulations, that is, without reweighting. Results are reported in Figure 4d. The quality of the fit is also summarized in the reported χ^2^ values. The addition of a simple torsional correction to the Aduri parameters results in a decrease in the overall χ^2^ from 15.23 to 9.17. However, this decrease is dominated by the χ^2^ of the A1 data point, which is reduced from χ^2^ = 84.64 to zero. Conversely, the χ^2^ averaged on all of the other experiments increases from χ^2^ = 7.57 to χ^2^ = 10.19. This indicates that including in the fitting the single A1 data point makes the agreement with denaturation experiments worse. On the other hand, the two sets of parameters obtained in this work (fit_A and fit_AB) display a significantly better agreement with experimental data. Note that fit_A, surprisingly, performs moderately better than fit_AB. The reason is that fit_AB, based on systems with double methylation and thus lower statistical efficiency, was performed with a higher regularization hyperparameter and thus parametrization closer to the reference one, as shown in Figures S6 and S7. Finally, as observed in the previous subsection, we note that the improvement in reproducing experiments is obtained by relatively small changes in the partial charges (see also Section S5). The fitted parameters are summarized in Table 2.

**Table 2:** Charge Modifications (*ΔQ*) and Torsional Potential (*V*_η_) for the Fitting Performed on the Smaller Data Set (fit_A, α = 10) and for the Fitting Performed on the Larger Data Set (fit_AB, α = 50)^a^

	C6 (e)	N6 (e)	H61 (e)	N1 (e)	C10 (e)	H100 (e)	*V*_η_ (kJ/mol)
fit_A	0.019	0.077	0.099	–0.046	0.004	–0.051	2.46
fit_AB	0.009	0.049	0.067	–0.053	0.033	–0.035	2.49

a For future simulations, we recommend using fit_A, which leads to a lower error on the larger set of available experiments.

### Relative Stability of *syn* and *anti* Conformations

One piece of the experimental information that we implicitly used in our fitting procedure is the relative stability of *syn* and *anti* conformations in a nucleotide. We indeed assumed a predominant population of *syn* conformation for the unpaired nucleotides used in the reference single-stranded systems. We also assumed that m^6^A adopts exclusively its *anti* conformation when paired, in agreement with experiments.^9,11^ In particular, ref (11) reports that, for the most common G6C sequence, m^6^A forms a Watson–Crick base pair with uridine that transiently exchanges on the millisecond time-scale between the main substate (*anti*) and a lowly populated (1%), singly hydrogen-bonded and mismatch-like conformation through isomerization of the methylamino group to the *syn* conformation. This population corresponds to a *ΔG*_*syn*/*anti*_^duplex^ ≈ −11 kJ/mol. We *a posteriori* validated this population by performing alchemical transformations on the duplex systems enforcing the *syn* conformation. The predicted *ΔG*_*syn*/*anti*_ values for a nucleotide and two of the tested duplexes are reported in Table 3, where the corresponding experimental values are also included. For the A1 experiment, as expected, the proposed sets of parameters closely match the experimental value that was used during training. The Aduri et al. force field underestimates the *ΔG*_*syn*/*anti*_, resulting in a relatively high population of the unexpected *anti* conformation in a nucleoside. This difference can be directly corrected with a torsional potential applied on the η torsion (Aduri+tors). However, when analyzing duplexes A2 and A3 with the Aduri+tors parameters, we found that the predicted *ΔG*_*syn*/*anti*_ would be close to zero, in fact resulting in the assumption of neglecting the *syn* conformation in duplexes in our alchemical calculations being difficult to justify, and in disagreement with experimental findings. In other words, the original Aduri charges allow reproduction of the relative stability of *syn* and *anti* conformations in either the paired state (Aduri parameters) or the unpaired state (with torsional correction), but not in both simultaneously. Remarkably, the sets of parameters proposed here, which also contain a torsional term penalizing the *anti* conformation, result in a significantly higher value for *ΔG*_*syn*/*anti*_^duplex^, much closer to a qualitative agreement with the experiment. This suggests that the proposed parameters better describe the interactions of the m^6^A nucleobase with the surrounding environment and are thus more transferable. We notice that the relative stability of *syn* and *anti* conformations is predicted to be sequence-dependent, being different for system A3 (sequence U6G).

**Table 3:** Free-Energy Differences between *syn* and *anti* Isomer States in Systems A1–A3^a^

	Aduri	Aduri+tors	fit_A	fit_AB	exp
(A1) *ΔG*_*syn*/*anti*_	1.71 ± 0.25	6.33 ± 0.25	6.07 ± 0.21	6.04 ± 0.26	6.3
(A2) *ΔG*_*syn*/*anti*_^duplex^	–7.7 ± 0.5	–3.1 ± 0.4	–10.4 ± 0.6	–7.8 ± 0.4	∼−11
(A3) *ΔG*_*syn*/*anti*_^duplex^	–5.4 ± 0.5	–0.8 ± 0.4	–4.9 ± 0.6	–5.8 ± 0.5	

a The last column corresponds to experimental estimates, whereas the other columns correspond to computed *ΔΔG* for different parametrizations. Energies are given in kJ/mol.

To gain insight into how the m^6^A–U pairings occur in the duplexes, we analyzed snapshots of system A2, for m^6^A in both *syn* and *anti*, together with histograms of distances between atoms belonging to the two nucleobases (Figure 5). The reported histograms are unimodal and with an increased average associated with the distortion of the A–U Watson–Crick pairings due to the steric clash induced by the methylation. However, the hydrogen bond between A–N1 and U–H3 is present, in agreement with what has been suggested previously.^11^

**Figure 5:**
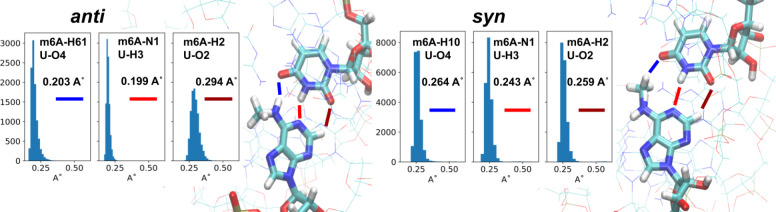
Interfacing atom distances for m^6^A–U pairing in system A2 in Table 1, for *anti* conformation (left) and *syn* (right). Histograms show unimodal distributions, and the averaged value are indicated in the box. Distances are sampled from the alchemical trajectories considering only the λ = 1 replica. In the *syn* conformation, m^6^A–H10 corresponds to the hydrogen of the methyl group closest to the uracil oxygen O4.

### Interpretation of Parameters and Dependence on Ionic Strength and Temperature

To provide an interpretation for the obtained parameters, we performed a few additional fittings. In particular, we investigated which charges have a major impact on enforcing agreement with experiments. For this purpose, we performed a fitting on exclusively two charges at a time plus the torsional term, considering pairs of charges that, in fit_A and fit_AB, have systematically positive and negative *ΔQ*s. Results are discussed in Section S7 and Figure S9. Overall, the results suggest that the main contribution of the fitted correction is to increase the stability of Watson–Crick hydrogen bonds by making N1 and H61 more polar and at the same time using the η torsional potential to control the *syn*/*anti* relative population.

As a further test, we simulated a subset of the systems using a salt concentration of 1 M, which is consistent with that used in experiments. As shown in Section S8, results are equivalent to those obtained at a 0.1 M salt concentration. These tests were performed using the fit_AB set of charges, but for one system, we repeated them with the original Aduri charges, with equivalent results. We interpret this result with the fact that the methylation is not sufficiently altering the electrostatic environment to be sensitive to changes in ion concentration. This implies that training using simulations performed at a different ion concentration would result in an equivalent set of parameters and further confirms the robustness of our results.

In addition, considering that the experimental results refer to a temperature *T* = 310 K, which is different from the simulation temperature (*T* = 300 K), we performed control simulations and analyses to assess the impact of this choice. Results are reported in Section S9 and show that such a temperature difference is not relevant for the results discussed here.

## Discussion

In this work, we proposed a protocol to parametrize charges in modified nucleobases using available melting experiments. The approach is applied to m^6^A and leads to a set of charges that can reproduce a set of 10 independent experimental values. The approach is based on the force-field fitting strategies introduced in earlier works,^27,51,52^ which are here extended with several technical improvements.

A first methodological contribution is a formalism that allows alchemical calculations to be used as a reference. Previous works were only using observables computed with a single set of force-field parameters.^14,27,51,52^ The method introduced here allows free-energy differences between different sets of parameters to be evaluated and compared with the experiment. This opens the way to the optimization of parameters based on experimentally measured *ΔΔG*s. We based our analysis on optical melting experiments, which are commonly employed in the nucleic acids community,^53^ but other types of experiments might be considered. In our specific application, only the parameters of one of the two end states were refined, but one could similarly fit parameters for both adenosine and m^6^A, at the price of increasing the number of parameters and thus the risk of overfitting. A second improvement is that we developed a way to efficiently recompute the total energy of the system using test charges. This is achieved by precomputing the total electrostatic energy of the system with a set of randomly perturbed charges. Given the high cost of electrostatic calculations, this makes the cost of each of the iterations performed during force-field fitting significantly faster and implicitly takes into account combination rules, nonbonded exclusions, and periodicity. These two improvements can be readily integrated into other MD-based force-field optimization strategies.

A limitation of optimizing charges with the introduced procedure is that the statistical efficiency of reweighting is significantly decreased even by small charge perturbations. This implies that simultaneously parametrizing many copies of the same nucleotide, or parametrizing a larger number of charges for the same nucleotide, would be more difficult. In our case, we had to include at most two m^6^A residues in the same simulation. If more copies of the same reparametrized nucleotide are needed in the same system, one might have to design strategies where only a few copies at a time are reparametrized, or follow an iterative procedure where modifications are included in consecutive steps.^27^ In this application, this was not necessary.

Overfitting was avoided by using a standard L2 regularization term on the charge increments. This penalty does not depend on the charge location. Importantly, the regularization hyperparameters tune the relative weight of the experimental data and of the reference charges, here taken from ref (21), thus allowing a meaningful set of parameters to be obtained also in regimes where the number of data points is very limited. It is worth noting that the standard restrained ESP fitting is performed including a restraint that acts as a hyperbolic regularization term,^50^ which is introduced to keep the absolute values of the obtained charges as small as possible. Our regularization, instead, keeps the resulting charges as close as possible to the initial guess obtained with the restrained ESP procedure.^21^ This allows the implicit inclusion in the fitting of the result of the corresponding quantum-mechanical calculation. More effective regularization strategies might be designed based on the molecular dipole, as done in ref (54), to minimize the perturbation of the electrostatic potential at a large distance from the molecule. Alternatively, one might directly use as a regularization term the deviation from the quantum-mechanical electrostatic potential at a short distance. In the limit of a large regularization hyperparameter, this would lead to ESP charges.^55^ Finally, other regularization criteria might be used.^14^ When comparing our procedure with standard ESP charge fitting, it is important to realize that we are aiming to reproduce experimentally observed *ΔΔG*s, which are nonlinear functions of the energy of each configuration, which in turn is a quadratic function of the charges. These nonlinearities make it possible for multiple local minima of the cost function to exist and could thus make the minimization not reproducible. However, when sufficiently regularized, the fitting procedure results in reproducible charges that depend smoothly on the control parameters. In standard ESP fitting, instead, the electrostatic potential is fitted, thus resulting in a linear fit with a unique solution.

We notice that the parameters of the unmethylated force field were not modified. This was based on the assumption that the employed set of force-field parameters is already capable of reproducing *ΔΔG* experiments associated with mutations between nonmodified nucleobases.^56^ The m^6^A charge optimization could be easily repeated using another set of initial parameters, and the parameters of nonmodified nucleobases might be adjusted as well, although with the caveat discussed above.

Another possible limitation of the employed alchemical simulations is the sufficient sampling of the end states. The duplex is expected to be stable and well structured, so sampling multiple structures should not be necessary. For selected cases, we also explored the possibility to include the unlikely *syn* paired state, which, as expected, gives a negligible contribution to the stability of the duplex. For single strands, instead, we only sampled the *syn* state. More importantly, our simulations were short enough to avoid any significant reconformation of the single strand. Sampling the conformations of flexible, single-stranded RNAs is notoriously difficult.^12^ In addition, the generated ensemble might contain artificially stabilized intercalated structures, whose population is known to be overestimated by the RNA and water force fields adopted here.^57,58^ This would make the correct sampling of the single-stranded state unfeasible. We also notice that the experimental results that we aimed to reproduce were performed on systems designed to have the isolated strands unstructured, to capture the effect of methylation on hybridization. Putting everything together, we conclude that the approximation of a single strand ensemble that does not depart too much from the initial A-form helix is a sensible choice for this specific application.

An important finding of this work is that the parameters of Aduri et al. cannot reproduce the *syn*/*anti* balance expected for m^6^A residues. This balance is extremely important and is related to the mechanism by which m^6^A modifications modulate duplex stability.^9^ This could not be rectified with a straightforward correction of the single torsion involved. The optimized charges, instead, allow the correct *syn*/*anti* balance to be recovered in both paired and unpaired nucleobases as well as a heterogeneous set of optical melting experiments to be reproduced. Interestingly, the Aduri et al. parameters were tested in a recent work,^18^ with results for system A2 in Table 1 consistent with ours and with experiments. However, systems A1 and A3 were not tested, and thus, the problem that we observed here could not be identified. Another interesting finding is that the *ΔΔG*s associated with N6 methylation are here predicted to be independent of ion concentration. We are not aware of any experimental validation of this finding, which could be obtained by comparing melting experiments at different ion concentrations. Finally, our results suggest that the relative population of the *syn* excited state in duplexes^11^ might significantly depend on the identity of the neighboring nucleotides. The precise hybridization kinetics could thus be quantitatively different for RNAs with different sequences.

A convenient property of our approach is that it does not require changing the functional form of the interaction potential so that new parameters can be readily incorporated into existing MD software. This is not the case if *ad hoc* corrections are employed.^14,59^ In addition, it is worth noting that the charge modifications obtained are very small, and in particular, they are smaller than the typical difference between sets of charges derived with slightly different procedures or using different reference conformations. Despite this small difference, the effect on experimental observables is significant. These observations imply that there is still significant space to improve the performance of current force fields without necessarily modifying the functional form if experimental information is used during training.^25^

Using our approach, it is possible to dissect the individual contribution of the modified force-field parameters. The main factors playing a role in the change of duplex stability induced by m^6^A methylation are (a) the penalty for switching to the unfavored *anti* isomer,^9^ (b) the stabilization induced by hydrophobic shielding of the methyl group against surrounding bases (see also Figure S8),^60,61^ (c) the impact of partial charges on stacking interactions,^60^ and (d) the impact on the strength of Watson–Crick hydrogen bonds. Since, on average, experimental *ΔΔG*s for denaturation experiments performed on duplexes are smaller than the *anti* isomer penalty, we could expect that the sum of the other factors has a stabilizing effect on the majority of the considered duplexes. We notice that Aduri charges for N1 and H61, which are involved in Watson–Crick pairings with the complementary uridine, have partial charge absolute values significantly lower compared to the standard adenine parameters (0.289 48 vs 0.411 50 for H61, – 0.675 968 vs −0.761 50 for N1). This may lead to a weakening of hydrogen bonds which may cause an overestimation of destabilization induced on duplexes, as we observed in Aduri+tors cases (see Figure 4d). The results of our fitting systematically increase the absolute value of H61 and N1 partial charges, hence resulting in a stronger Watson–Crick pairing. At the same time, the torsional term allows a reproduction of the correct *anti* isomer penalty. Parameters are coupled so that it is necessary to fit them simultaneously to avoid double counting effects.

To the best of our knowledge, this is the first attempt to tune partial charges of a biomolecular force field based on experiments performed on macromolecular complexes. We expect that this approach could be used in the future to improve the capability of biomolecular force fields to match experimental observations by exploiting a part of the functional form that has been traditionally derived in a bottom-up fashion. For future work, we recommend using the fit_A set of parameters (parameters available at https://github.com/bussilab/m6a-charge-fitting), which leads to a lower error on the larger set of available experiments. Remarkably, the parameters derived here for m^6^A allow a proper description of paired and unpaired m^6^A in both *syn* and *anti* conformations and thus open the way to the use of molecular simulations to quantitatively investigate the effects of N6 methylations on RNA structural dynamics.

## Supporting Information Available

Eleven sections discussing (1) initial tests performed with Aduri parameters; (2) λ interpolation and spacing; (3) details on weight calculation; (4) details in cross validation; (5) all sets of tested charges; (6) details on the fitting procedure; (7) tests optimizing two charges at a time; (8) results at a higher ionic concentration; (9) results at a higher temperature; (10) list of simulations; and (11) structural snapshots (PDF)

## Notes

*Data Availability*. Jupyter notebooks used for molecular dynamics simulations and analysis and parameter files for m6A can be found at https://github.com/bussilab/m6a-charge-fitting. Input files and trajectory data are available at DOI 10.5281/zenodo.6498020.

## References

[ref1] Gilbert W. V.; Bell T. A.; Schaening C. Messenger RNA modifications: form, distribution, and function. Science 2016, 352, 1408–1412. 10.1126/science.aad8711. 27313037 PMC5094196

[ref2] Harcourt E. M.; Kietrys A. M.; Kool E. T. Chemical and structural effects of base modifications in messenger RNA. Nature 2017, 541, 339–346. 10.1038/nature21351. 28102265 PMC5498787

[ref3] Patil D. P.; Chen C.-K.; Pickering B. F.; Chow A.; Jackson C.; Guttman M.; Jaffrey S. R. m6A RNA methylation promotes XIST-mediated transcriptional repression. Nature 2016, 537, 369–373. 10.1038/nature19342. 27602518 PMC5509218

[ref4] He P. C.; He C. m6A RNA methylation: from mechanisms to therapeutic potential. EMBO J. 2021, 40, e105977 10.15252/embj.2020105977. 33470439 PMC7849164

[ref5] Tanzer A.; Hofacker I. L.; Lorenz R. RNA modifications in structure prediction-Status quo and future challenges. Methods 2019, 156, 32–39. 10.1016/j.ymeth.2018.10.019. 30385321

[ref6] Liu N.; Dai Q.; Zheng G.; He C.; Parisien M.; Pan T. N 6-methyladenosine-dependent RNA structural switches regulate RNA-protein interactions. Nature 2015, 518, 560–564. 10.1038/nature14234. 25719671 PMC4355918

[ref7] Huang L.; Ashraf S.; Wang J.; Lilley D. M. Control of box C/D snoRNP assembly by N6-methylation of adenine. EMBO Rep. 2017, 18, 1631–1645. 10.15252/embr.201743967. 28623187 PMC5579392

[ref8] Jones A. N.; Tikhaia E.; Mourão A.; Sattler M. Structural effects of m6A modification of the Xist A-repeat AUCG tetraloop and its recognition by YTHDC1. Nucleic Acids Res. 2022, 50, 2350–2362. 10.1093/nar/gkac080. 35166835 PMC8887474

[ref9] Roost C.; Lynch S. R.; Batista P. J.; Qu K.; Chang H. Y.; Kool E. T. Structure and thermodynamics of N6-methyladenosine in RNA: a spring-loaded base modification. J. Am. Chem. Soc. 2015, 137, 2107–2115. 10.1021/ja513080v. 25611135 PMC4405242

[ref10] Hopfinger M. C.; Kirkpatrick C. C.; Znosko B. M. Predictions and analyses of RNA nearest neighbor parameters for modified nucleotides. Nucleic Acids Res. 2020, 48, 8901–8913. 10.1093/nar/gkaa654. 32810273 PMC7498315

[ref11] Liu B.; Shi H.; Rangadurai A.; Nussbaumer F.; Chu C.-C.; Erharter K. A.; Case D. A.; Kreutz C.; Al-Hashimi H. M. A quantitative model predicts how m6A reshapes the kinetic landscape of nucleic acid hybridization and conformational transitions. Nat. Commun. 2021, 12, 5201 10.1038/s41467-021-25253-8. 34465779 PMC8408185

[ref12] Šponer J.; Bussi G.; Krepl M.; Banáš P.; Bottaro S.; Cunha R. A.; Gil-Ley A.; Pinamonti G.; Poblete S.; Jurečka P.; Walter N. G.; Otyepka M. RNA structural dynamics as captured by molecular simulations: a comprehensive overview. Chem. Rev. 2018, 118, 4177–4338. 10.1021/acs.chemrev.7b00427. 29297679 PMC5920944

[ref13] Tan D.; Piana S.; Dirks R. M.; Shaw D. E. RNA force field with accuracy comparable to state-of-the-art protein force fields. Proc. Natl. Acad. Sci. U.S.A. 2018, 115, E1346–E1355. 10.1073/pnas.1713027115. 29378935 PMC5816156

[ref14] Fröhlking T.; Mlỳnskỳ V.; Janeček M.; Kührová P.; Krepl M.; Banáš P.; Šponer J.; Bussi G. Automatic learning of hydrogen-bond fixes in an AMBER RNA force field. J. Chem. Theory Comput. 2022, 18, 4490–4502. 10.1021/acs.jctc.2c00200. 35699952 PMC9281393

[ref15] Xu Y.; Vanommeslaeghe K.; Aleksandrov A.; MacKerell A. D. Jr; Nilsson L. Additive CHARMM force field for naturally occurring modified ribonucleotides. J. Comput. Chem. 2016, 37, 896–912. 10.1002/jcc.24307. 26841080 PMC4801715

[ref16] Li Y.; Bedi R. K.; Wiedmer L.; Huang D.; Sledz P.; Caflisch A. Flexible binding of m6A reader protein YTHDC1 to its preferred RNA motif. J. Chem. Theory Comput. 2019, 15, 7004–7014. 10.1021/acs.jctc.9b00987. 31670957

[ref17] Li Y.; Bedi R. K.; Wiedmer L.; Sun X.; Huang D.; Caflisch A. Atomistic and Thermodynamic Analysis of N6-Methyladenosine (m6A) Recognition by the Reader Domain of YTHDC1. J. Chem. Theory Comput. 2021, 17, 1240–1249. 10.1021/acs.jctc.0c01136. 33472367

[ref18] Hurst T.; Chen S.-J. Deciphering nucleotide modification-induced structure and stability changes. RNA Biol. 2021, 18, 1920–1930. 10.1080/15476286.2021.1882179. 33586616 PMC8583165

[ref19] Krepl M.; Damberger F. F.; von Schroetter C.; Theler D.; Pokorná P.; Allain F. H.-T.; vSponer J. Recognition of N6-Methyladenosine by the YTHDC1 YTH Domain Studied by Molecular Dynamics and NMR Spectroscopy: The Role of Hydration. J. Phys. Chem. B 2021, 125, 7691–7705. 10.1021/acs.jpcb.1c03541. 34258996

[ref20] Purslow J. A.; Nguyen T. T.; Khatiwada B.; Singh A.; Venditti V. N 6-methyladenosine binding induces a metal-centered rearrangement that activates the human RNA demethylase Alkbh5. Sci. Adv. 2021, 7, eabi8215 10.1126/sciadv.abi8215. 34407931 PMC8373141

[ref21] Aduri R.; Psciuk B. T.; Saro P.; Taniga H.; Schlegel H. B.; SantaLucia J. AMBER force field parameters for the naturally occurring modified nucleosides in RNA. J. Chem. Theory Comput. 2007, 3, 1464–1475. 10.1021/ct600329w. 26633217

[ref22] Stasiewicz J.; Mukherjee S.; Nithin C.; Bujnicki J. M. QRNAS: software tool for refinement of nucleic acid structures. BMC Struct. Biol. 2019, 19, 1–11. 10.1186/s12900-019-0103-1. 30898165 PMC6429776

[ref23] Kierzek E.; Kierzek R. The thermodynamic stability of RNA duplexes and hairpins containing N6-alkyladenosines and 2-methylthio-N6-alkyladenosines. Nucleic Acids Res. 2003, 31, 4472–4480. 10.1093/nar/gkg633. 12888507 PMC169893

[ref24] Kierzek E.; Zhang X.; Watson R. M.; Kennedy S. D.; Szabat M.; Kierzek R.; Mathews D. H. Secondary structure prediction for RNA sequences including N6-methyladenosine. Nat. Commun. 2022, 13, 1–10. 10.1038/s41467-022-28817-4. 35277476 PMC8917230

[ref25] Fröhlking T.; Bernetti M.; Calonaci N.; Bussi G. Toward empirical force fields that match experimental observables. J. Chem. Phys. 2020, 152, 230902 10.1063/5.0011346. 32571067

[ref26] Mey A. S.; Allen B. K.; Macdonald H. E. B.; Chodera J. D.; Hahn D. F.; Kuhn M.; Michel J.; Mobley D. L.; Naden L. N.; Prasad S.; et al. Best Practices for Alchemical Free Energy Calculations [Article v1.0]. Living J. Comp. Mol. Sci. 2020, 2, 18378 10.33011/livecoms.2.1.18378. PMC8388617 34458687

[ref27] Cesari A.; Bottaro S.; Lindorff-Larsen K.; Banáš P.; ŠPoner J.; Bussi G. Fitting corrections to an RNA force field using experimental data. J. Chem. Theory Comput. 2019, 15, 3425–3431. 10.1021/acs.jctc.9b00206. 31050905

[ref28] Alenaizan A.; Barnett J. L.; Hud N. V.; Sherrill C. D.; Petrov A. S. The proto-Nucleic Acid Builder: a software tool for constructing nucleic acid analogs. Nucleic Acids Res. 2021, 49, 79–89. 10.1093/nar/gkaa1159. 33300028 PMC7797056

[ref29] Abraham M. J.; Murtola T.; Schulz R.; Páll S.; Smith J. C.; Hess B.; Lindahl E. GROMACS: High performance molecular simulations through multi-level parallelism from laptops to supercomputers. SoftwareX 2015, 1, 19–25. 10.1016/j.softx.2015.06.001.

[ref30] Bernetti M.; Bussi G. Pressure control using stochastic cell rescaling. J. Chem. Phys. 2020, 153, 114107 10.1063/5.0020514. 32962386

[ref31] Cornell W. D.; Cieplak P.; Bayly C. I.; Gould I. R.; Merz K. M.; Ferguson D. M.; Spellmeyer D. C.; Fox T.; Caldwell J. W.; Kollman P. A. A second generation force field for the simulation of proteins, nucleic acids, and organic molecules. J. Am. Chem. Soc. 1995, 117, 5179–5197. 10.1021/ja00124a002.

[ref32] Pérez A.; Marchán I.; Svozil D.; vSponer J.; Cheatham T. E. III; Laughton C. A.; Orozco M. Refinement of the AMBER force field for nucleic acids: improving the description of α/γ conformers. Biophys. J. 2007, 92, 3817–3829. 10.1529/biophysj.106.097782. 17351000 PMC1868997

[ref33] Zgarbová M.; Otyepka M.; Šponer J.; Mládek A.; Banáš P.; Cheatham T. E.; Jurečka P. Refinement of the Cornell et al. nucleic acids force field based on reference quantum chemical calculations of glycosidic torsion profiles. J. Chem. Theory Comput. 2011, 7, 2886–2902. 10.1021/ct200162x. 21921995 PMC3171997

[ref34] Jorgensen W. L.; Chandrasekhar J.; Madura J. D.; Impey R. W.; Klein M. L. Comparison of simple potential functions for simulating liquid water. J. Chem. Phys. 1983, 79, 926–935. 10.1063/1.445869.

[ref35] Joung I. S.; Cheatham T. E. III Determination of alkali and halide monovalent ion parameters for use in explicitly solvated biomolecular simulations. J. Phys. Chem. B 2008, 112, 9020–9041. 10.1021/jp8001614. 18593145 PMC2652252

[ref36] Beutler T. C.; Mark A. E.; van Schaik R. C.; Gerber P. R.; Van Gunsteren W. F. Avoiding singularities and numerical instabilities in free energy calculations based on molecular simulations. Chem. Phys. Lett. 1994, 222, 529–539. 10.1016/0009-2614(94)00397-1.

[ref37] Goga N.; Rzepiela A.; de Vries A.; Marrink S.; Berendsen H. Efficient algorithms for Langevin and DPD dynamics. J. Chem. Theory Comput. 2012, 8, 3637–3649. 10.1021/ct3000876. 26593009

[ref38] Parrinello M.; Rahman A. Polymorphic transitions in single crystals: A new molecular dynamics method. J. Appl. Phys. 1981, 52, 7182–7190. 10.1063/1.328693.

[ref39] Darden T.; York D.; Pedersen L. Particle mesh Ewald: An N log (N) method for Ewald sums in large systems. J. Chem. Phys. 1993, 98, 10089–10092. 10.1063/1.464397.

[ref40] Souaille M.; Roux B. Extension to the weighted histogram analysis method: combining umbrella sampling with free energy calculations. Comput. Phys. Commun. 2001, 135, 40–57. 10.1016/S0010-4655(00)00215-0.

[ref41] Shirts M. R.; Chodera J. D. Statistically optimal analysis of samples from multiple equilibrium states. J. Chem. Phys. 2008, 129, 124105 10.1063/1.2978177. 19045004 PMC2671659

[ref42] Tan Z.; Gallicchio E.; Lapelosa M.; Levy R. M. Theory of binless multi-state free energy estimation with applications to protein-ligand binding. J. Chem. Phys. 2012, 136, 144102 10.1063/1.3701175. 22502496 PMC3339880

[ref43] Efron B.; Tibshirani R. Bootstrap methods for standard errors, confidence intervals, and other measures of statistical accuracy. Stat. Sci. 1986, 54–75. 10.1214/ss/1177013815.

[ref44] Bennett C. H. Efficient estimation of free energy differences from Monte Carlo data. J. Comput. Phys. 1976, 22, 245–268. 10.1016/0021-9991(76)90078-4.

[ref45] Wu D.; Kofke A. D. Phase-space overlap measures. II. Design and implementation of staging methods for free-energy calculations. J. Chem. Phys. 2005, 123, 084109 10.1063/1.2011391. 16164284

[ref46] Zhu C.; Byrd R. H.; Lu P.; Nocedal J. Algorithm 778: L-BFGS-B: Fortran subroutines for large-scale bound-constrained optimization. ACM Trans. Math. Softw. 1997, 23, 550–560. 10.1145/279232.279236.

[ref47] Virtanen P.; Gommers R.; Oliphant T. E.; Haberland M.; Reddy T.; Cournapeau D.; Burovski E.; Peterson P.; Weckesser W.; Bright J.; et al. SciPy 1.0: fundamental algorithms for scientific computing in Python. Nat. Methods 2020, 17, 261–272. 10.1038/s41592-019-0686-2. 32015543 PMC7056644

[ref48] Kish L. Survey sampling; John Wiley & Sons: New York, 1965.

[ref49] Rangan R.; Bonomi M.; Heller G. T.; Cesari A.; Bussi G.; Vendruscolo M. Determination of structural ensembles of proteins: restraining vs reweighting. J. Chem. Theory Comput. 2018, 14, 6632–6641. 10.1021/acs.jctc.8b00738. 30428663

[ref50] Cieplak P.; Cornell W. D.; Bayly C.; Kollman P. A. Application of the multimolecule and multiconformational RESP methodology to biopolymers: Charge derivation for DNA, RNA, and proteins. J. Comput. Chem. 1995, 16, 1357–1377. 10.1002/jcc.540161106.

[ref51] Norgaard A. B.; Ferkinghoff-Borg J.; Lindorff-Larsen K. Experimental parameterization of an energy function for the simulation of unfolded proteins. Biophys. J. 2008, 94, 182–192. 10.1529/biophysj.107.108241. 17827232 PMC2134871

[ref52] Li D.-W.; Brüschweiler R. Iterative optimization of molecular mechanics force fields from NMR data of full-length proteins. J. Chem. Theory Comput. 2011, 7, 1773–1782. 10.1021/ct200094b. 26596440

[ref53] Schroeder S. J.; Turner D. H. Meth. Enzymol.; Elsevier, 2009; Vol. 468; pp 371–387. 10.1016/S0076-6879(09)68017-4 PMC4070882 20946778

[ref54] Janeček M.; Kührová P.; Mlỳnskỳ V.; Otyepka M.; ŠPoner J.; Banáš P. W-RESP: Well-restrained electrostatic potential-derived charges. Revisiting the charge derivation model. J. Chem. Theory Comput. 2021, 17, 3495–3509. 10.1021/acs.jctc.0c00976. 33999623

[ref55] Singh U. C.; Kollman P. A. An approach to computing electrostatic charges for molecules. J. Comput. Chem. 1984, 5, 129–145. 10.1002/jcc.540050204.

[ref56] Sakuraba S.; Asai K.; Kameda T. Predicting RNA Duplex Dimerization Free-Energy Changes upon Mutations Using Molecular Dynamics Simulations. J. Phys. Chem. Lett. 2015, 6, 4348–4351. 10.1021/acs.jpclett.5b01984. 26722970

[ref57] Condon D. E.; Kennedy S. D.; Mort B. C.; Kierzek R.; Yildirim I.; Turner D. H. Stacking in RNA: NMR of four tetramers benchmark molecular dynamics. J. Chem. Theory Comput. 2015, 11, 2729–2742. 10.1021/ct501025q. 26082675 PMC4463549

[ref58] Bottaro S.; Bussi G.; Kennedy S. D.; Turner D. H.; Lindorff-Larsen K. Conformational ensembles of RNA oligonucleotides from integrating NMR and molecular simulations. Sci. Adv. 2018, 4, eaar8521 10.1126/sciadv.aar8521. 29795785 PMC5959319

[ref59] Kührová P.; Mlỳnskỳ V.; Zgarbová M.; Krepl M.; Bussi G.; Best R. B.; Otyepka M.; Šponer J.; Banáš P. Improving the performance of the AMBER RNA force field by tuning the hydrogen-bonding interactions. J. Chem. Theory Comput. 2019, 15, 3288–3305. 10.1021/acs.jctc.8b00955. 30896943 PMC7491206

[ref60] Guckian K. M.; Schweitzer B. A.; Ren R. X.-F.; Sheils C. J.; Tahmassebi D. C.; Kool E. T. Factors contributing to aromatic stacking in water: evaluation in the context of DNA. J. Am. Chem. Soc. 2000, 122, 2213–2222. 10.1021/ja9934854. 20865137 PMC2943206

[ref61] Isaksson J.; Chattopadhyaya J. A uniform mechanism correlating dangling-end stabilization and stacking geometry. Biochemistry 2005, 44, 5390–5401. 10.1021/bi047414f. 15807532

