# Supplementary material for: Molecular Simulations Matching Denaturation Experiments for N6-Methyladenosine
Source: ACS Cent Sci. 2022 Aug 3;8(8):1218–28. doi: 10.1021/acscentsci.2c00565 (PMC9413829; doi:10.1021/acscentsci.2c00565)
Supplement: Supplementary file 1 — oc2c00565_si_001.pdf [file oc2c00565_si_001.pdf]

Supporting Information for: Molecular simulations matching denaturation experiments for N6-methyladenosine

Valerio Piomponi[1], Thorben Fröhlking[1], Mattia Bernetti[1], and Giovanni Bussi[1*]

[1]*Scuola Internazionale Superiore di Studi Avanzati, SISSA, via Bonomea 265, 34136 Trieste, Italy*

E-mail: bussi@sissa.it

Supporting information contains:

- 35 pages.
- 11 sections.
- 18 figures.
- 10 tables.

S1 Parameters adapted from Aduri et al.

Before reparametrizing charges using alchemical calculations, we adapted the Aduri et al. parameters[1] to the current AMBER force field.[2–4] In particular, we used backbone charges from the current AMBER force field combined with the original Aduri et al. parameters, adding a correction spread on all atoms so as to preserve the correct charge of the nucleotide. Charges are reported in Table S2. We then considered the parametrization of the angle $\eta 6$, defined by the atoms N1-C6-N6-C10. We performed a well-tempered metadynamics calculation[5] using $\eta 6$ as a collective variable. Metadynamics was performed using the PLUMED package,[6] with a simulation length of 100 *ns*, depositing a Gaussian every 500 time steps, with initial height equal to 1.2 *kJ/mol* and width $\sigma = 0.35$. The Bias factor was set to 10. We then used the free energy profile computed along $\eta 6$ (see Fig. S1) to estimate the $\Delta G_{syn/anti}$ by integrating over the two corresponding minima. The result is $\Delta G_{syn/anti} = 1.5$ *kJ/mol*, which is an underestimation with respect to the experimental value 6.3 *kJ/mol*. We thus decided to add to the potential a single torsional term in this form

$$U(x_i) = \frac{V_\eta}{2}[1 + cos(\eta_6(x_i) - \pi)] \tag{1}$$

For positive value of the parameter V_η this correction penalizes the *anti* conformations. We then used the MetaD trajectory and obtained biases in a reweighting scheme, tuning the parameter V_η in order to enforce the experimental value of $\Delta G_{syn/anti}$. Specifically, we assigned a weight $w(x)$ to each frame, computed as

$$w(x) \propto e^{\beta B(\eta_6(x))} e^{-\beta \frac{V_\eta}{2}[1+cos(\eta_6(x)-\pi)]} \tag{2}$$

Here $B(\eta_6)$ is the bias potential constructed during MetaD simulation and dependent on time.

The $\Delta G_{syn/anti}$ was then obtained as

$$\Delta G_{syn/anti} = -\frac{1}{\beta}\log(\frac{\sum_{x\in syn} w(x)}{\sum_{x\in anti} w(x)}) \tag{3}$$

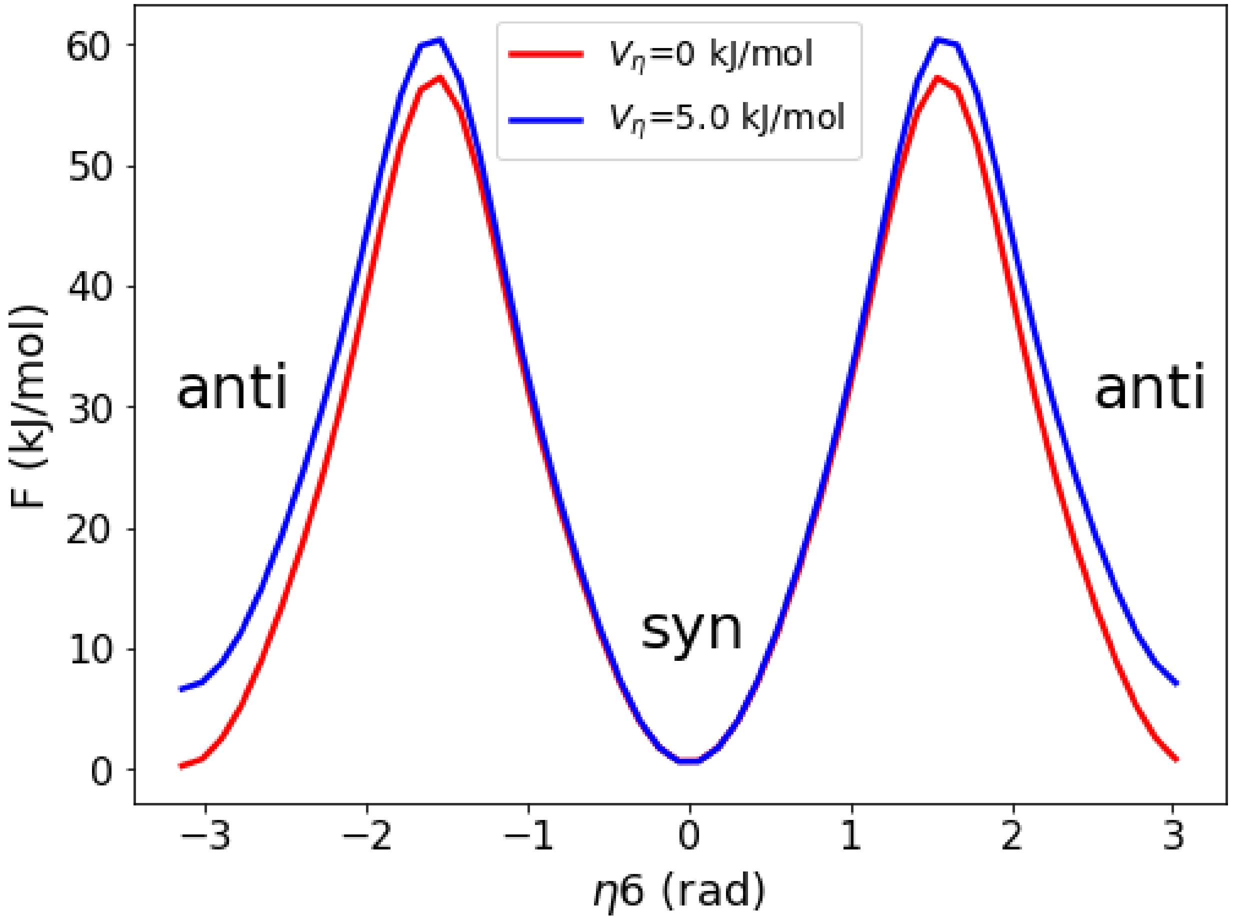

Figure S1: Free Energy Profiles along $\eta 6$ reconstructed using Metadynamics along the collective variable $\eta 6$. The red line corresponds to the profile obtained with the standard Aduri parametrization ($V_\eta = 0\ kJ/mol$), whereas the blue line corresponds to the one obtained with Aduri+tors parametrization ($V_\eta = 5\ kJ/mol$)

We iteratively adjusted V_η until we found that $V_\eta = 5.0\ kJ/mol$ results in a $\Delta G_{syn/anti} = 6.4 \pm 0.3\ kJ/mol$, which is compatible with experiment. Statistical error was computed using block analysis.[5] Figure S1 shows the Free Energy profiles reconstructed along $\eta 6$ for $V_\eta = 0$ (reference) and $V_\eta = 5.0\ kJ/mol$.

S2 λ Spacing

An important choice in setting up alchemical free-energy calculations (AFECs) is the number of intermediate steps and the optimal form of the intermediate Hamiltonian functions. In the GROMACS implementation we used, soft core potentials are used to interpolate Lennard-Jones and Coulomb potentials as follows

$$V_{sc,\lambda}(r) = (1-\lambda)V_A((\alpha\sigma^6\lambda + r^6)^{\frac{1}{6}}) + \lambda V_{m^6A}((\alpha\sigma^6(1-\lambda)^6 + r^6)^{\frac{1}{6}}) \tag{4}$$

where $\alpha = 0.5$ and $\sigma = 0.3$ nm. Here, V_A and V_{m^6A} the Lennard-Jones and Coulomb potential energy functions for unmodified and modified adenine, respectively, $V_{sc,\lambda}$ is the interpolated version of the function, and r the interatomic distance. The energy of the only scaled torsion (η_6) is instead a linear combination of the energy of the two end points with factors $1-\lambda$ and λ, and is defined as

$$V_{\eta_6,\lambda}(x) = (1-\lambda)V_{\eta_6,A} + \lambda V_{\eta_6,m^6A} \tag{5}$$

In our case, $\lambda = 0$ denotes the parameters of the unmodified adenine, whereas $\lambda = 1$ those of the modified adenine.

We decided not to scale the bonded interactions that are present in only one of the systems (e.g., torsional parameters associated to the methyl group), but to rather have a single H in one of the topologies and a CH_3 group in the other topology. These groups are present in both systems, though with their nonbonded interactions switched off at one of the end points. This implies that the intermediate topologies contain both C10 and H62. Also the torsional potentials controlling the rotation of the amino group in the unmodified nucleotide and of the carbon in the modified nucleotide are not scaled. These potentials are symmetric with respect to *syn*/*anti* rotations, and thus do not influence the *syn*/*anti* population. We instead scaled the potential acting on η_6 since this torsional potential is

not symmetric and its presence would lead to a *syn*/*anti* balance different from zero in the unmodified nucleotide. Readers interested in reproducing this setup are encouraged to inspect the GROMACS topology files provided in the Zenodo archive.

We also notice that GROMACS allows setting separate scaling factors for electrostatic, Lennard-Jones, and bonded interactions. We didn't exploit this feature, and rather scaled both electrostatic and Lennard-Jones interactions with the same λ factor. This still leaves the open issue of placing a suffient number of λ factors interpolating between 0.0 and 1.0. In a replica-exchange setting, the acceptance rate can be used as a measure of the phase-space overlap between adjacent ensembles. A minimum acceptance is then required to enable mixing of ensembles. At the same time, the spacing in λ required to reach this minimum acceptance might differ in different regions of the λ space, thus leading to an optimal allocation of replicas that is not uniformly spaced in λ. We here used a single system, that is the *stand alone* nucleoside in solution, to optimize this ladder and then reused the same parameters for all systems. Specifically, we empirically adjusted the λ values until we obtained a set of 16 intermediates (set1 in Table S1) leading to an approximately uniform acceptance rate (Figure S2), each of them greater than 20%. As it can be seen, the density of the chosen λ values is inhomogenous and, in particular, higher close to the boundaries ($\lambda = 0$ or 1). This set of lambdas was then used for all the AFECs presented in this work where a single adenine is methylated, and as expected lead to an acceptance greater than 20% for most replica pairs, and greater than 10% for all replica pairs. In cases where two methylations were included (systems B2 to B5 in Table 1 of main text), we found that for some pairs of replicas the acceptance was significantly lower than 20%. We notice that in principle the presence of two simultaneous methylation should lead to a larger number of replicas required to obtain the same acceptance. We notice however that some of the acceptances reported in Fig. S2 are significantly larger than 20%. By reoptimizing the parameters, we obtained a set of 16 λ's (set 2 in Table S1) that was able to guarantee an acceptance greater than 20% for all transitions in systems with two methylations.

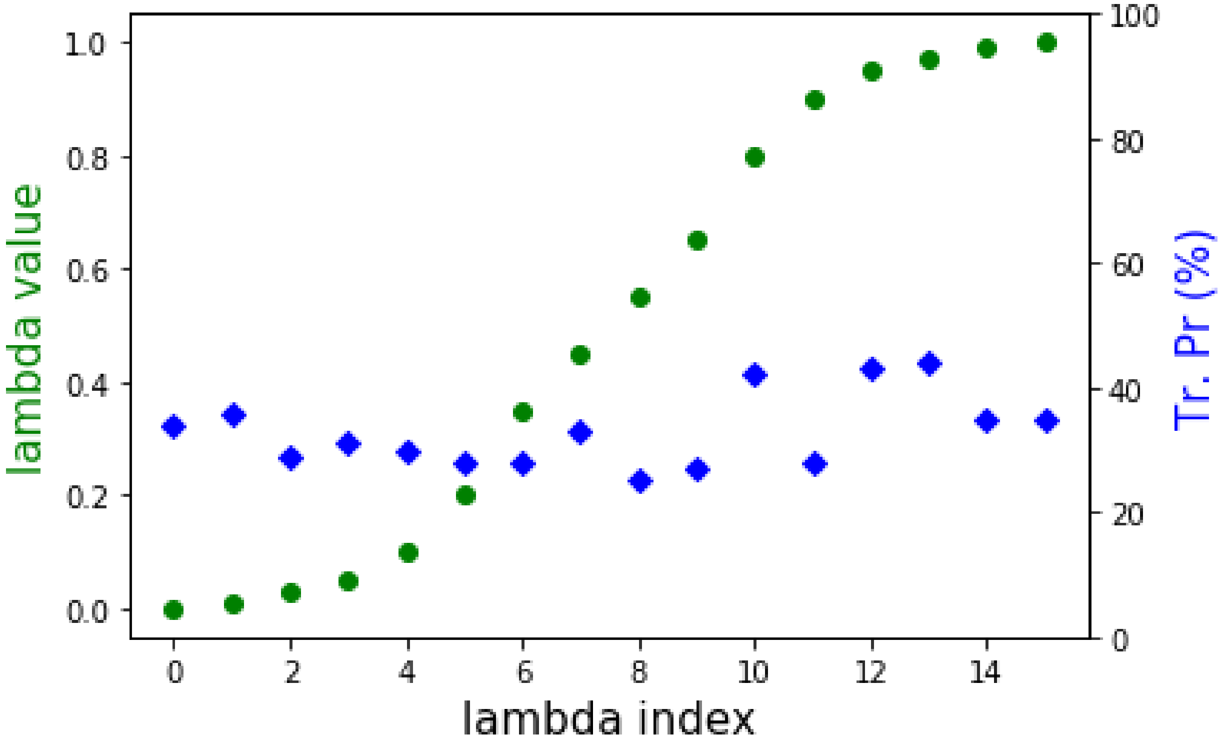

Figure S2: set1 λ values (green circles) and transition probalities in HREX between replica λ_i and λ_{i+1}(blue squares) as a function of λ index i.

Table S1: Sets of lambda coefficients used in AFEC replica exchange simulations for systems with a single methylation (set 1) or with two methylations (set 2).

	0	1	2	3	4	5	6	7	8	9	10	11	12	13	14	15
**set1**	0.0	0.01	0.03	0.05	0.10	0.2	0.35	0.45	0.55	0.65	0.8	0.9	0.95	0.97	0.99	1.0
**set2**	0.0	0.02	0.05	0.09	0.14	0.2	0.3	0.43	0.57	0.7	0.8	0.86	0.91	0.95	0.98	1.0

S3 Weight calculation

A crucial step in the analysis of the alchemical calculations is the evaluation of weights. We consider a set of N trajectories obtained using different value of λ, so that λ_k denotes the value of λ and n_k the number of snapshots in the k-th simulation. The k-th trajectory will thus contain samples from the distribution $P_k(x) \propto e^{-\beta E_{\lambda_k}(x)}$, where $E_\lambda(x)$ is the energy associated conformation x for a given λ and β is the inverse of the thermal energy. We are interested in obtaining weights $w(x)$ that can be used to compute averages corresponding to a reference value of λ ($\lambda = 0$). In other words, for any observable $O(x)$, its average at $\lambda = 0$ is obtained as

$$\langle O \rangle = \sum_x w(x) O(x) \tag{6}$$

where the sum runs over the concatenation of the N trajectories. By using the WHAM method in its binless formulation,[7–9] the unnormalized weights can be obtained as:

$$w(x) \propto \frac{1}{\sum_{k=1}^{N} n_k e^{-\beta(E_{\lambda_k}(x) - E_0(x))} Z_k^{-1}} \tag{7}$$

and subsequently normalized scaling them by a factor ensuring that $\sum_x w(x) = 1$. The partition function associated with each value of k, Z_k, can be obtained as

$$Z_k = \sum_x w(x) e^{-\beta(E_{\lambda_k}(x) - E_0(x))} \tag{8}$$

These two equations should be solved self-consistently. For numerical purposes, it is convenient to initially remove from the computed energies ($E_{\lambda_k}(x)$) their minimum along both k and x so as to avoid numerical overflows in the calculation of the exponential function, and then add the corresponding contributions to the resulting free energies and to the logaritm of the weights. The calculation was performed using the wham tool available in the bussilab python package, which can be obtained at `https://github.com/bussilab/py-bussilab`, version 0.0.36, and that can be used as a reference for the exact numerical procedure used

here. Once the weights have been obtained, they can be used to compute the free-energy difference between the two end states using Eq. (1) is the main text.

Notably, the weights only depend on the conformation (x) and not on the specific value of λ at which the conformation was generated. This implies that trajectories can be concatenated in any order resulting in identical weights. This allows to concatenate "demuxed" (continuous) trajectories, which are virtually independent of each other, being coupled only through the exchange step. By performing a blocked bootstrap with block size identical to the length of each trajectory, one ensures that correlations are minimized. In this case, however, one should explicitly take into account that, for a given bootstrap sample, the number of snapshots generated at each value of λ will differ.

The notebook `AFEC_example/AFEC_computations.ipynb` in repository `https://github.com/bussilab/m6a-charge-fitting` reports the calculation of the free-energy change associated with the conversion of an adenosine nucleoside to m^{6}A and can be used to perform the analysis using the BAR method and the binless WHAM method, and then to compute statistical errors using blocked bootstrap.

S4 Cross validation details

We here report the results of the cross-validation procedure as a function of the hyper-parameter α (regularization on charges), by keeping the hyper-parameter $\beta = 0$ (regularization on torsional potential). Results for the fitting on the smaller dataset (fit_A) are reported in Fig. S3. Results for the fitting on the larger dataset (fit_AB) are reported in Fig. S4.

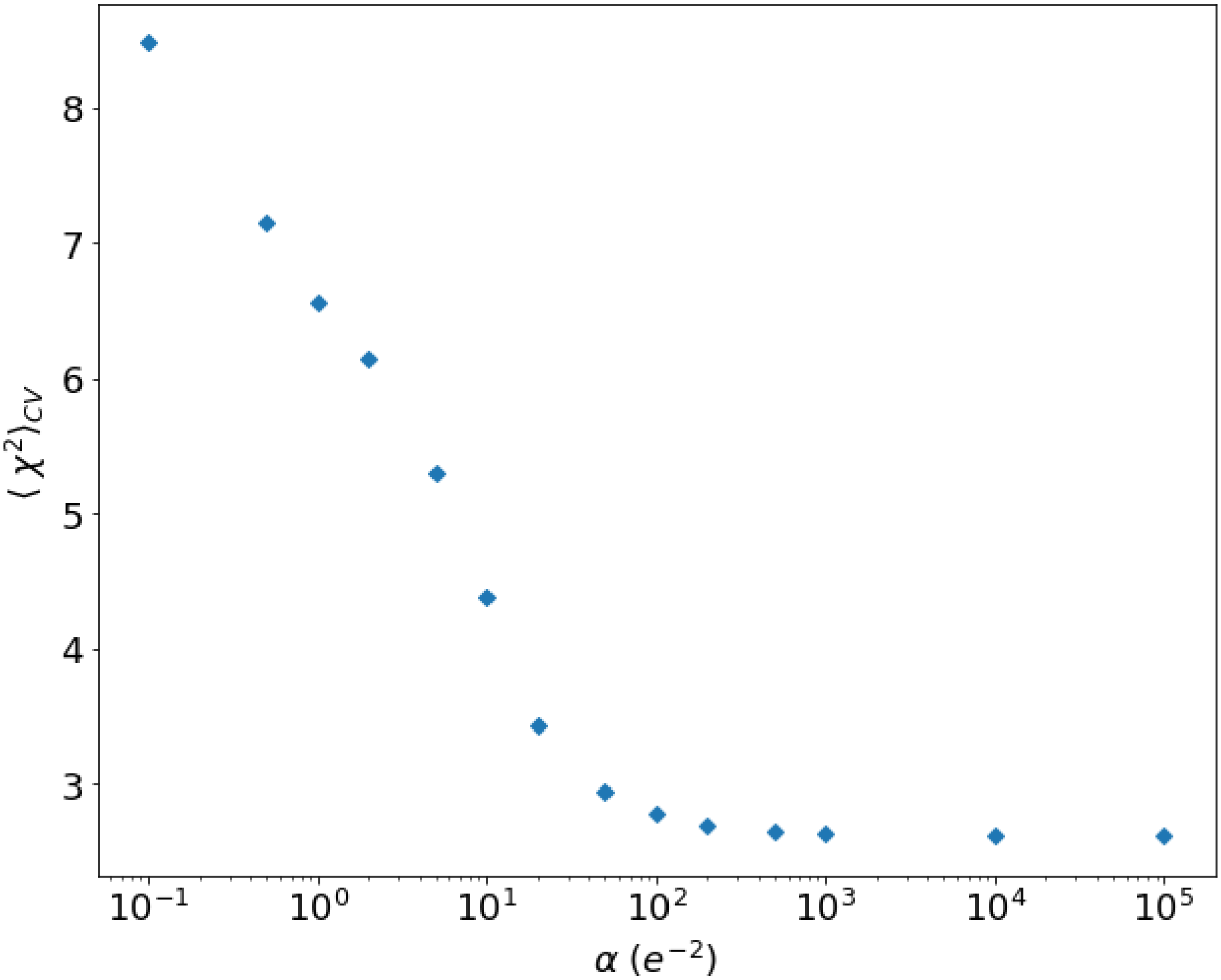

Figure S3: Cross Validation with Leave One Out precedure in fit_A. Projection of data in plot of Fig. 3a in main text along $\beta = 0$.

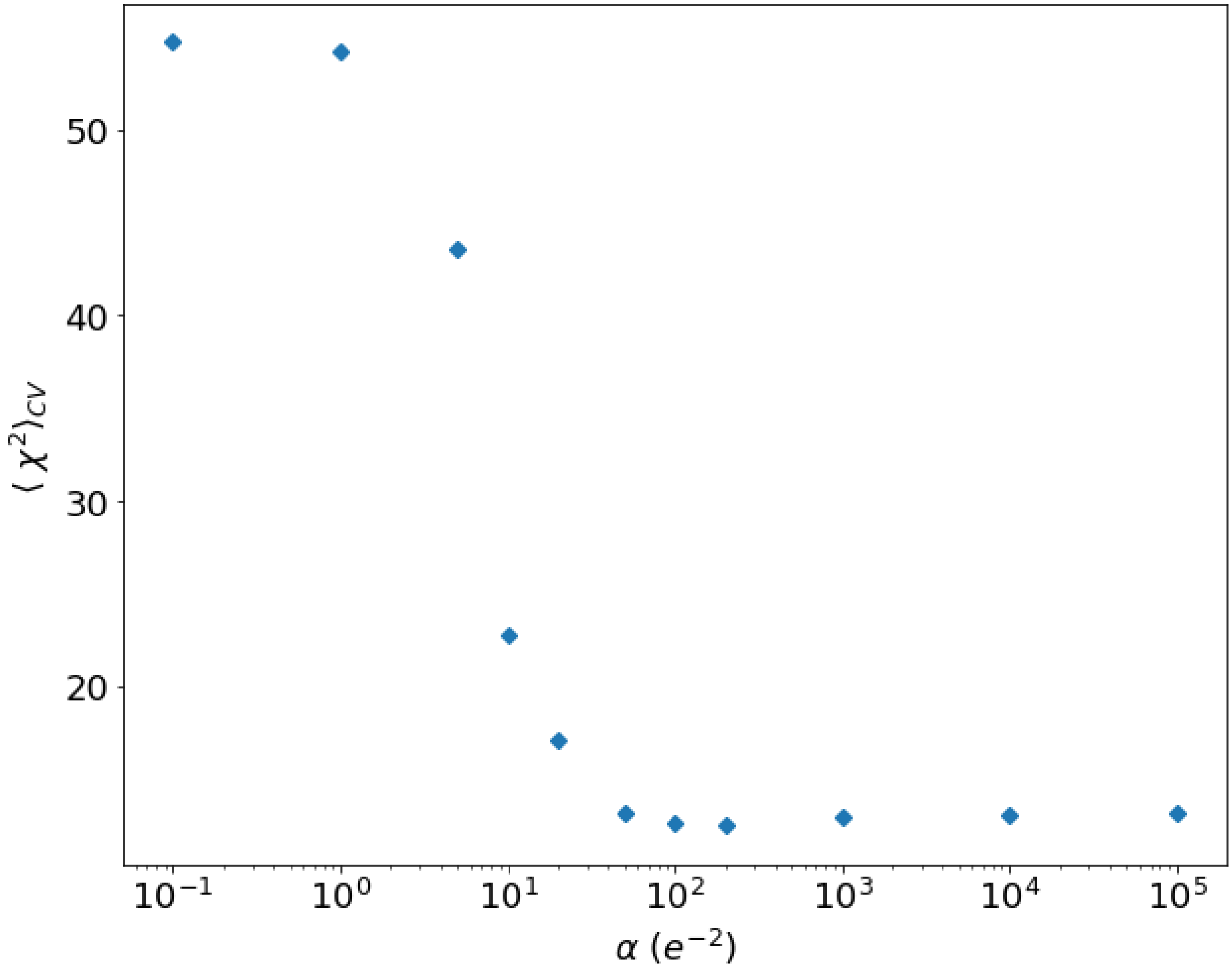

Figure S4: Cross Validation with Leave One Out precedure in fit_AB. Projection of data in plot of Fig. 4a in main text along $\beta = 0$.

S5 Sets of charges

Table S2: Charges for all atoms of the m6A nucleobase for different parametrizations.

	**Aduri**	**fit_A**	**fit_AB**	**RESP_anti**	**RESP_syn**	**Krepl**
**N9**	-0.07829	-0.07829	-0.07829	-0.0564	-0.1834	-0.1719
**C8**	0.13844	0.13844	0.13844	0.0815	0.2573	0.0631
**H8**	0.16681	0.16681	0.16681	0.1726	0.1329	0.1973
**N7**	-0.59080	-0.59080	-0.59080	-0.5250	-0.5854	-0.5652
**C5**	0.03544	0.03544	0.03544	0.0226	-0.2346	0.0152
**C6**	0.44911	0.46851	0.45811	0.5880	0.7140	0.5597
**N6**	-0.30623	-0.22923	-0.25723	-0.3756	-0.4189	-0.4756
**H61**	0.28948	0.38888	0.35648	0.3306	0.3392	0.3232
**C10**	-0.28897	-0.28467	-0.25597	-0.3009	-0.3239	-0.0774
**H101**	0.12596	0.07536	0.09096	0.1299	0.1400	0.0774
**H102**	0.12596	0.07536	0.09096	0.1299	0.1400	0.0774
**H103**	0.12596	0.07536	0.09096	0.1299	0.1400	0.0774
**N1**	-0.67597	-0.72167	-0.72897	-0.8746	-0.7617	-0.6604
**C2**	0.55132	0.55132	0.55132	0.6898	0.5688	0.4636
**H2**	0.05539	0.05539	0.05539	0.0485	0.0692	0.0865
**N3**	-0.73497	-0.73497	-0.73497	-0.8037	-0.7900	-0.7027
**C4**	0.48723	0.48723	0.48723	0.4807	0.6559	0.4589

Table S2 shows all the sets of charges considered for the m6A nucleobase. The first column corresponds to the charges from Aduri et al.,[1] adjusted to be compatible with the current AMBER force field.[2–4] Namely, we used the standard AMBER charges for backbone and sugar, and the Aduri et al. charges for the nucleobase. We subtracted 0.0003 e from the charge of each atom so as to maintain the neutral charge of the nucleoside. The following columns represent the charges obtained in our fittings, using: the regularized fitting (with $\alpha = 10$) on set A, fit_A; the regularized fitting (with $\alpha = 50$) on set AB, fit_AB. In addition, we show charges that we derived following the standard procedure on a nucleobase. We here considered the geometry of both isomers (*syn* and *anti*), computed the electrostatic potentials of the N6-methylated adenine base by Gaussian 09[10] using the HF/6-31G* level of theory,

subsequently deriving the partial charges via the RESP method.[11] For these calculations we replaced the sugar with a closing methyl group, as done by Aduri *et al.* We notice however that Aduri *et al.* does not report the chosen isomer, which is likely a *syn*, the most populated one for an isolated nucleobase. The last set of parameters (Krepl) have been used in Ref.[12] and were kindly shared by Miroslav Krepl. In order to visualize these sets of charges in a dimensional reduced space, we perform principle components analysis (PCA) on the charges data set, both considering the entire nucleobase (Fig. S5) or only the 6 charges involved in the fitting (Fig. S6). As it can be appreciated in the PCA analysis, the difference by the charges resulting from our fitting procedures and those reported by Aduri is very small, and significantly lower than the typical variability between different sets of charges obtained with slighly different procedure.

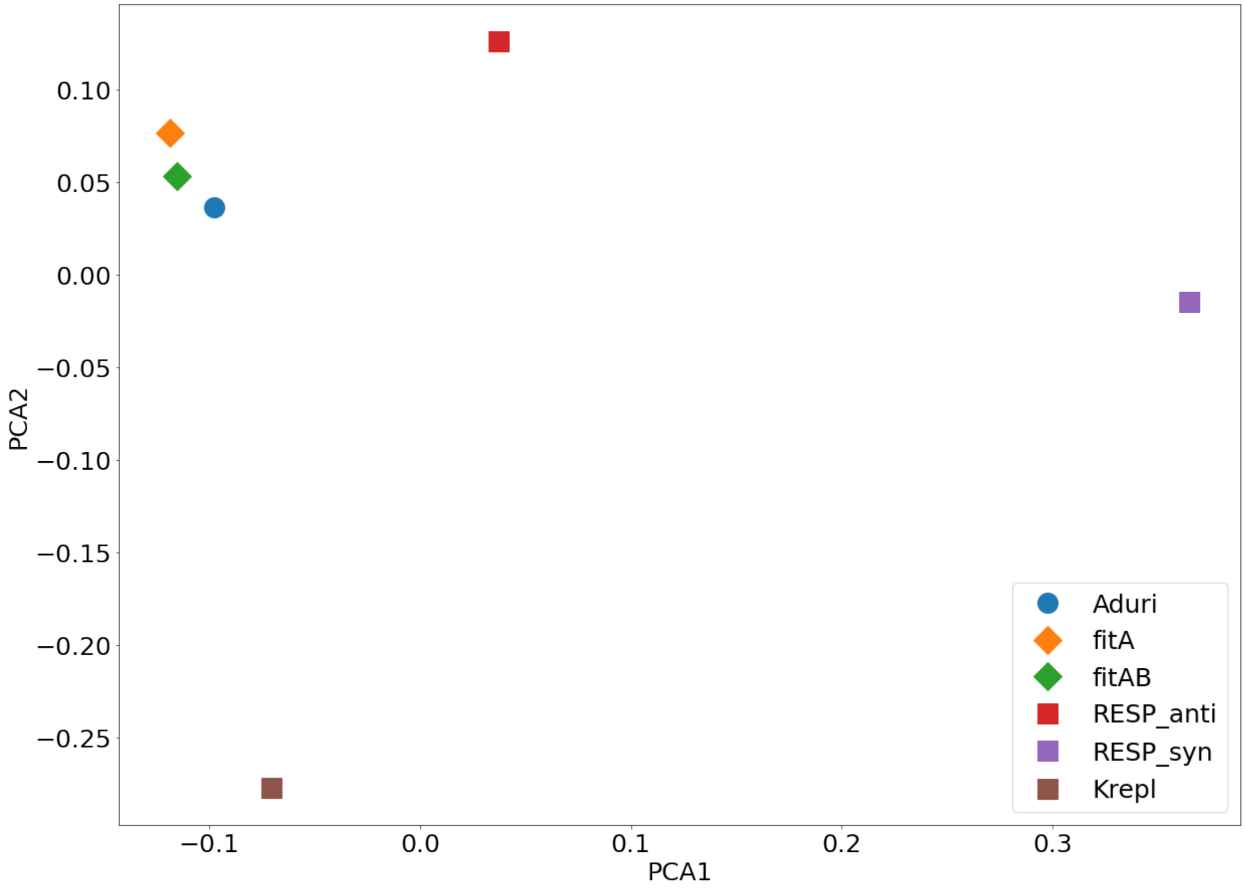

Figure S5: PCA performed giving as input all the charges of the nucleobase.

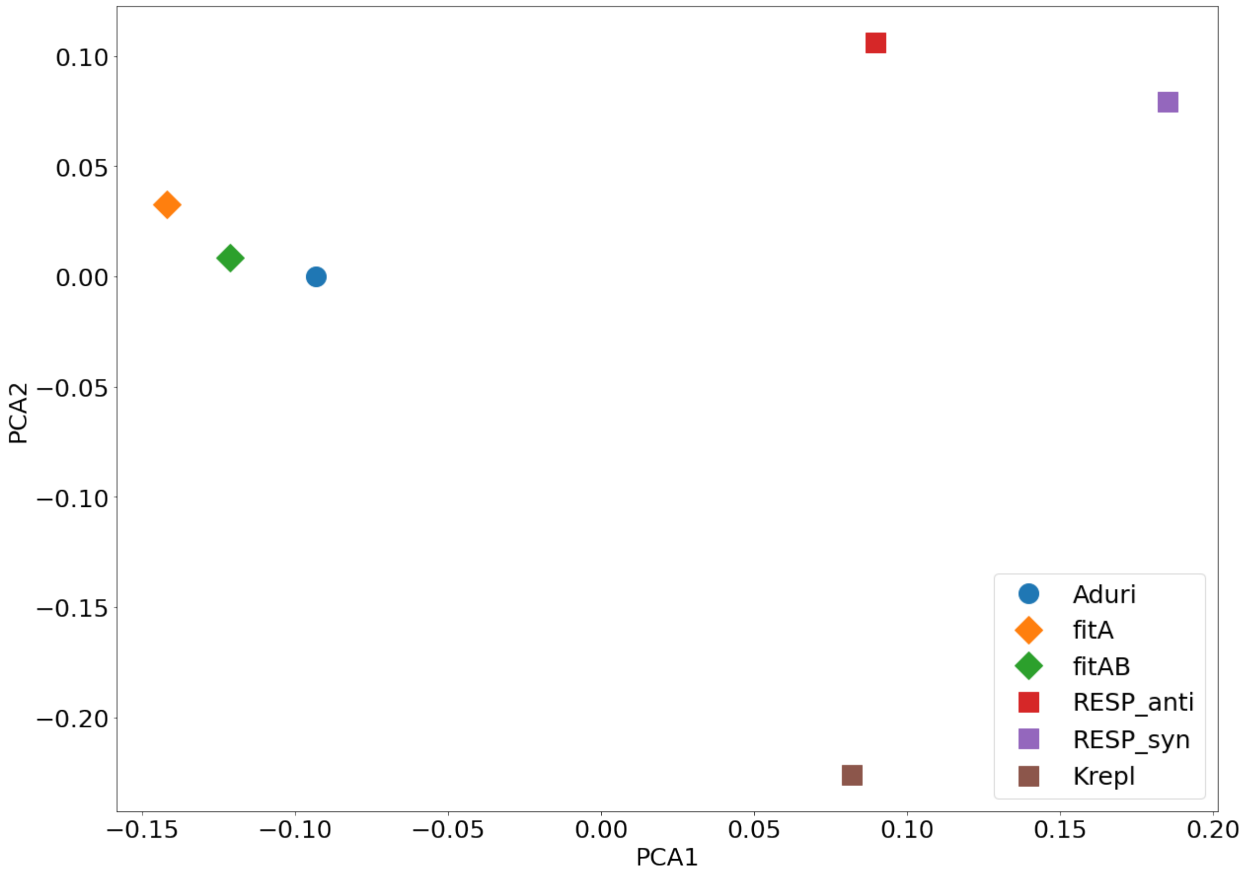

Figure S6: PCA performed giving as input only the 6 charges involved in the fitting.

S6 Formulas used in the fitting procedure

Our fitting is based on the minimization of a cost function defined as:

$$C = \chi^2 + \alpha \sum_{i=0}^{5} \Delta Q_i^2 + \beta V_\eta^2 = \chi^2 + \alpha[\sum_{i=1}^{5} \Delta Q_i^2 + (\sum_{i=1}^{5} \Delta Q_i)^2] + \beta V_\eta^2 \tag{9}$$

where α and β are the hyperparameters needed for regularization of the charges and the torsional term respectively. Here we assumed that $\Delta Q_0 = -\sum_{i=1}^{5} \Delta Q_i$, to preserve the total charge. The χ^2 measures the discrepancy between computations and experiments:

$$\chi^2 = \frac{1}{N_{exp}} \sum_{i=1}^{N_{exp}} \frac{(\Delta G_{2i-1} - \Delta G_{2i} - \Delta\Delta G_i^{exp})^2}{\sigma_i^2} \tag{10}$$

Alchemical ΔGs are computed through a reweighting procedure via the equation:

$$\Delta G^{AFEC} = -k_B T \log(\frac{\sum_i^{N_{frame}} w_i e^{-\beta[\Delta E(x_i) + \Delta U(x_i)]}}{\sum_i^{N_{frame}} w_i}) \tag{11}$$

where w_i are the weight derived by the binless WHAM on the original set of energies. ΔU correspond to variation in the potential in $\lambda = 1$ case due to modification of the fitted charges and torsional term:

$$\Delta U(x) = \sum_{i=5}^{5} K_i(x)\Delta Q_i + \sum_{i=1}^{5}\sum_{j=i}^{5} K_{ij}(x)\Delta Q_i \Delta Q_j + V_\eta[1 + cos(\eta_6(x_i) - \pi)] \tag{12}$$

and ΔE is:

$$\Delta E(x_i) = E_{\lambda=1}(x_i) - E_{\lambda=0}(x_i) \tag{13}$$

For the minimization of the cost function through the L-BFGS-B method,[13] the derivative of the cost function with respect to the fitted parameters is needed. This should be computed for charges as follows:

$$\frac{\partial C}{\partial \Delta \mathbf{Q}} = \frac{\partial C}{\partial \Delta \mathbf{G}} \frac{\partial \Delta \mathbf{G}}{\partial \mathbf{L}} \frac{\partial \mathbf{L}}{\partial \Delta \mathbf{Q}} \tag{14}$$

Here we introduced the 20-components vector

$$\mathbf{L} = (\Delta Q_1, \Delta Q_2, ..., \Delta Q_1 \Delta Q_1, \Delta Q_1 \Delta Q_2, ..., \Delta Q_5 \Delta Q_5) \tag{15}$$

For the torsional parameter instead we have:

$$\frac{\partial C}{\partial V_\eta} = \frac{\partial C}{\partial \Delta \mathbf{G}} \frac{\partial \Delta \mathbf{G}}{\partial V_\eta} \tag{16}$$

The derivative of the free-energy change with respect to $\mathbf{L}$ components can be computed as

$$\frac{\partial \Delta G_k}{\partial L_l} = \langle K_l \rangle_k = \sum_i^{N_{frame}} w_i K_l^i e^{-\beta[\Delta E(x_i) + \Delta U(x_i) + V_\eta [1 + cos(\eta_6(x_i) - \pi)]]} \tag{17}$$

The derivative of the free-energy change with respect to the torsional parameter can be computed as

$$\frac{\partial \Delta G_k}{\partial V_\eta} = \langle [1 + cos(\eta_6(x_i) - \pi)] \rangle_k = \sum_i^{N_{frame}} w_i [1 + cos(\eta_6(x_i) - \pi)] e^{-\beta[\Delta E(x_i) + \Delta U(x_i) + V_\eta [1 + cos(\eta_6(x_i) - \pi)]]} \tag{18}$$

Table S3: ΔGs computed through alchemical computations, with different parametrizations and Free Energy methods, reported in kJ/mol. We note that, in addition to the systems required to compute the *syn/anti* balance in the nucleoside (A1) and the effect of methylation in hybridization energies (A2–A5 and B1–B5), this table also reports control results for systems A2 and A3 where the duplex simulation was performed in the unexpected *syn* conformation.

	**Aduri**		**Aduri+tors**	**fit_A**		**fit_AB**	
method	BAR	WHAM	WHAM+tors	BAR	WHAM	BAR	WHAM
A1 *syn*	258.24 ± 0.22	258.24 ± 0.21	258.28 ± 0.21	207.19 ± 0.16	207.22 ± 0.16	211.42 ± 0.21	211.23 ± 0.18
A1 *anti*	260.12 ± 0.12	259.95 ± 0.15	264.61 ± 0.15	213.12 ± 0.20	213.29 ± 0.14	217.03 ± 0.13	217.27 ± 0.19
A2 dup *anti*	258.85 ± 0.70	258.63 ± 0.33	263.29 ± 0.33	208.32 ± 0.17	208.3 ± 0.5	214.3 ± 0.7	214.01 ± 0.35
A2 dup *syn*	266.44 ± 0.42	266.4 ± 0.4	266.4 ± 0.4	218.69 ± 0.29	218.74 ± 0.31	223.0 ± 0.5	222.98 ± 0.34
A2 ss *syn*	257.52 ± 0.31	257.54 ± 0.27	257.58 ± 0.27	206.45 ± 0.28	206.50 ± 0.29	210.34 ± 0.34	210.40 ± 0.22
A3 dup *anti*	261.56 ± 0.35	261.39 ± 0.32	266.15 ± 0.32	213.10 ± 0.44	213.0 ± 0.6	216.38 ± 0.43	216.21 ± 0.38
A3 dup *syn*	267.77 ± 0.35	267.75 ± 0.30	267.79 ± 0.30	217.78 ± 0.26	217.93 ± 0.24	221.96 ± 0.23	222.11 ± 0.27
A3 ss *syn*	257.75 ± 0.24	257.80 ± 0.32	257.84 ± 0.32	206.37 ± 0.29	206.31 ± 0.35	211.9 ± 0.5	211.17 ± 0.29
A4 dup *syn*	255.06 ± 0.19	255.07 ± 0.17	255.11 ± 0.17	203.76 ± 0.14	203.64 ± 0.19	208.11 ± 0.15	208.07 ± 0.17
A4 ss *syn*	257.40 ± 0.19	257.42 ± 0.18	257.46 ± 0.18	206.76 ± 0.18	206.70 ± 0.17	210.56 ± 0.13	210.62 ± 0.19
A5 dup *syn*	256.89 ± 0.11	256.80 ± 0.19	261.76 ± 0.19	206.06 ± 0.25	205.89 ± 0.16	209.88 ± 0.16	209.93 ± 0.16
A5 ss *syn*	257.56 ± 0.15	257.70 ± 0.23	257.74 ± 0.23	206.65 ± 0.15	206.68 ± 0.19	211.01 ± 0.18	210.96 ± 0.16
B1 dup *anti*	259.09 ± 0.30	259.18 ± 0.21	263.94 ± 0.21	209.67 ± 0.30	209.62 ± 0.38	213.79 ± 0.35	213.81 ± 0.22
B1 ss *syn*	257.73 ± 0.25	257.60 ± 0.36	257.64 ± 0.36	205.46 ± 0.14	205.37 ± 0.34	210.27 ± 0.39	210.3 ± 0.6
B2 dup *anti*	521.6 ± 0.9	521.6 ± 0.9	530.9 ± 0.9	425.24 ± 1.9	425.26 ± 1.3	434.2 ± 1.0	434.1 ± 1.2
B2 ss *syn*	258.34 ± 0.31	258.20 ± 0.35	258.24 ± 0.35	207.3 ± 0.5	206.3 ± 0.5	211.13 ± 0.40	211.19 ± 0.29
B3 dup *anti*	518.5 ± 1.0	518.6 ± 0.9	527.9 ± 0.9	420.7 ± 1.0	420.7 ± 0.9	430.6 ± 1.4	430.5 ± 0.5
B3 ss *syn*	257.74 ± 0.16	257.72 ± 0.27	257.76 ± 0.27	206.77 ± 0.23	206.75 ± 0.39	210.38 ± 0.35	210.45 ± 0.22
B4 dup *anti*	523.2 ± 1.2	523.1 ± 0.5	532.4 ± 0.5	428.8 ± 1.2	428.9 ± 0.9	433.6 ± 0.9	434.8 ± 1.1
B4 ss *syn*	257.84 ± 0.45	257.85 ± 0.41	257.89 ± 0.41	206.53 ± 0.29	206.72 ± 0.35	210.87 ± 0.35	210.7 ± 0.5
B5 dup *anti*	521.9 ± 0.8	522.5 ± 0.7	531.8 ± 0.7	424.0 ± 0.7	424.0 ± 1.1	434.4 ± 0.9	434.4 ± 1.1
B5 ss *syn*	257.04 ± 0.34	257.10 ± 0.43	257.14 ± 0.43	205.42 ± 0.25	205.37 ± 0.23	209.82 ± 0.35	209.85 ± 0.19

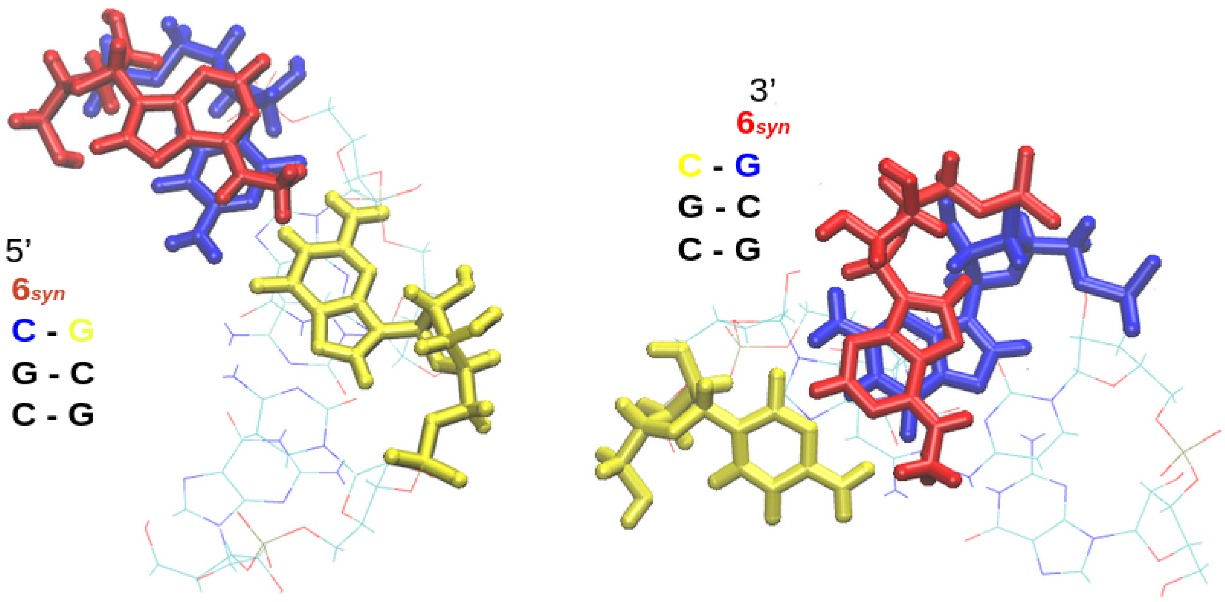

Figure S7: Snapshots from simulations of systems A4 (left) and A5 (right). These are the case where m6A appears as a dangling end and has a stabilization effect on the duplex inducing a favorable stacking. This is due to the hydrophobic screening of the methyl group against adjacent nucleobases. In the A4 case the stabilization effect is greater ($\Delta\Delta G \sim 2$ kJ/mol) and the methyl group appears in a conformation in which it is covering both neighboring bases from the top, whereas in the A5 case the stabilization is lower ($\Delta\Delta G \sim 1$ kJ/mol) and the methyl group appears to be shifted to the side.

S7 Fitting pairs of charges

In order to investigate the significance of the fitted parameters, we performed further fittings by tuning only subsets of the parameters. We notice that Aduri charges for N1 and H61, which are involved in Watson Crick parings with the paired uridine, have partial charge absolute value significatively lower compared to the standard adenine parameters (0.28948 vs 0.41150 for H61, -0.675968 vs. -0.76150 for N1). This may lead to a weakening of hydrogen bonds which may cause an overestimation of destabilization induced on duplexes, as we observed in Aduri+tors cases (see Figure 4d in main text). The results of our fitting systematically increase the absolute value of H61 ad N1 partial charges, hence resulting in a stronger Watson Crick pairing. At the same time, the torsional term allow to reproduce the correct *anti* isomer penalty. Parameters are coupled, so that it is necessary to fit them simultaneously so as to avoid double counting effects. To demonstrate that N1 and H61 are the most important charges to tune in order to reproduce experiments by strengthening hydrogen bonds, we performed 4 further fittings on the entire data set (AB) by tuning only the torsional plus 2 charges at time, respectively for the pairs N1-H61; N1-N6; H61-H101/2/3; N6-H101/2/3, which are taking into account atoms that have systematic positive and negative ΔQ both in fit_A and fit_AB. Results are summarized in Figure S8.

Interestingly, when fitting only 2 charges, the results are converging for α going to zero. Furthermore, the Kish Size Ratio obtained for $\alpha = 0$ is always greater than 0.18, demonstrating that statistical significance is always maintained when fitting only two charges. For α and β set to zero, results of the fitting are summarized in Table S4.

The lowest χ^2 is obtained in H61-N1 case with a value of 4.42 (for comparsion, the fitted χ^2 obtained in fit_AB is 3.61), confirming the hypothesis that tuning these two charges is crucial to reproduce experiments. A slightly larger χ^2 is obtained in N6-N1 case. Figure S8 also shows that the correction on the torsional angle is highly coupled with modifications of N1 charge. In the two tested cases where N1 was not fitted, the torsional parameter V_η has a smaller dependence on the charges.

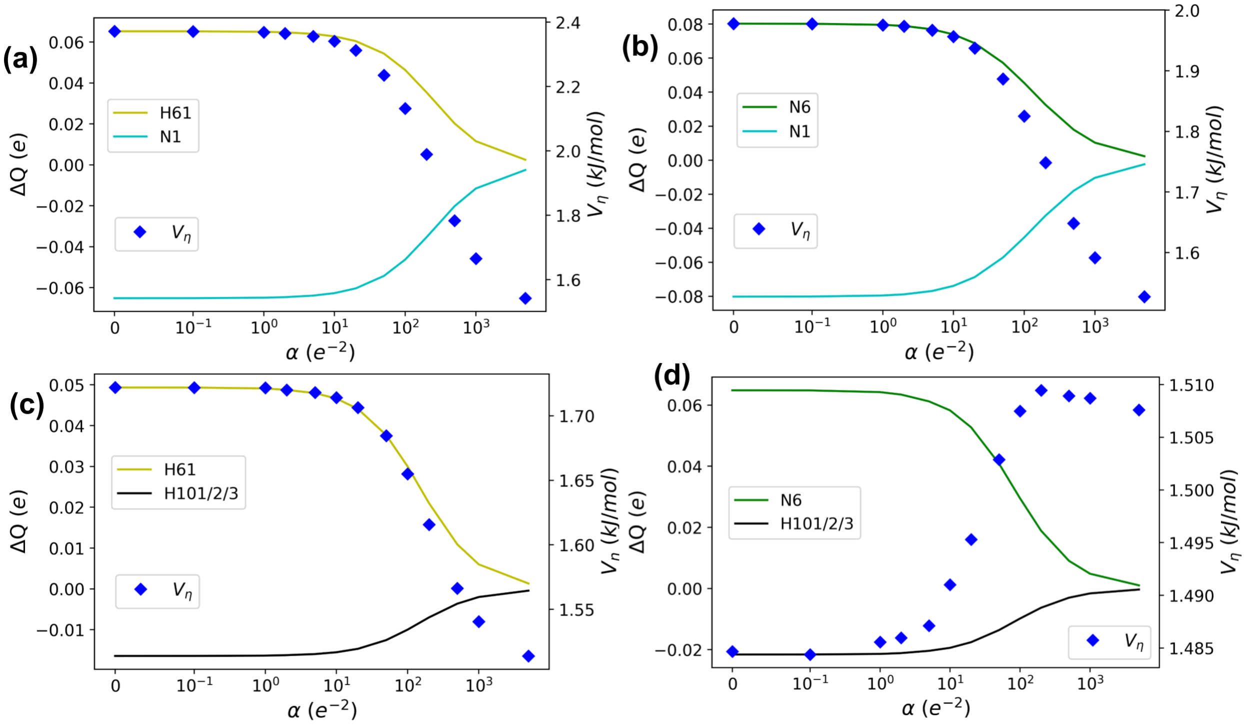

Figure S8: Fitted charges and torsional term V_η as a function of α ($\beta = 0$). Horizontal axes are in log scale except for 0–0.1 sections which are linear.

Table S4: Result for fitting 2 charges plus the torsional with hyperparameters set to 0. Only the ΔQ associated to the first atom is shown (H61 or N6). The ΔQ associated to N1 has the same absolute value and opposite sign. The H100 charge is equally distributed on the 3 hydrogens of the methyl group, so that the charge on each hydrogen has 1/3 absolute value and opposite sign when compared with the reported ΔQ.

	ΔQ (e)	V_η (kJ/mol)	χ^2	**KSR**
**H61-N1**	0.0652	2.37	4.42	0.18
**N6-N1**	0.0802	1.98	4.52	0.31
**H61-H100**	0.04932	1.72	5.92	0.54
**N6-H100**	0.0648	1.49	5.93	0.74

S8 Simulations at higher ionic concentration

In this work, the simulations used in the fitting were performed at a ionic concentration of 0.1 M NaCl, which is a value commonly used in molecular dynamics simulations. However, the standard condition in which denaturation experiments, inclusing those analyzed in this work, are performed is 1 M NaCl. In order to quantify the dependence of the computed $\Delta\Delta Gs$ on the ion concentration, we performed further control simulations for a subset of systems at 1 M NaCl. Systems A1, A2 and A4 were chosen in order involve in this checking all possible environments for the methyl group: a nucleoside, where the methyl group is isolated; a duplex with internal m6A, where the methyl group is partly hidden from interactions with ions; a duplex with m6A as a dangling end, where the methyl group is more exposed to interactions with ions; and the corresponding single stranded RNAs, so as to be able to obtain the $\Delta\Delta Gs$. For the nucleoside, the methylation was added in both *syn* and *anti* conformations. For the other systems, the methylation was added in the expected conformation, as we did for all other systems (see main text).

Results are summarized in Tables S5 and S6. The A1 system, that is the single nucletoide in solution, reports a shift in the ΔGs of about 1.2 kJ/mol with respect to 0.1 M cases, for both *syn* and *anti*. As a result, the relative $\Delta\Delta G$ is not affected. For all other system, the ΔGs for the two ionic concentration are in agreement within their statistical error. These results indicate that the fitting is not affected by the discrepancy between the ionic concentration used in computations and experiments.

Table S5: ΔGs computed through alchemical computations and binless WHAM method. Each row corresponds to a different NaCl ionic concentrations used in the simulation. Each column correspond so a different system. In the last column, results obtained with the original Aduri parameters are shown as well for one of the systems, confirming that the mild dependence on ion parameters is independent of the precise partial charges used in the simulation.

	**fit_AB**						**Aduri**
[NaCl]	**A1 syn**	**A1 anti**	**A2 dup**	**A2 ss**	**A4 dup**	**A4 ss**	**A2 ss**
0.1 M	211.23 ± 0.18	217.27 ± 0.19	214.01 ± 0.35	210.40 ± 0.22	208.07 ± 0.17	210.62 ± 0.19	257.54 ± 0.27
1 M	212.33 ± 0.36	218.46 ± 0.25	213.99 ± 0.25	210.53 ± 0.42	208.30 ± 0.33	210.91 ± 0.26	257.56 ± 0.26

Table S6: $\Delta\Delta G$s computed through alchemical computations and binless WHAM method. The first two rows correspond to different NaCl ionic concentrations used in the simulation, and last row corresponds to the reference experimental values.

[NaCl]	**A1**	**A2**	**A4**
0.1 M	6.04 ± 0.26	3.6 ± 0.4	-2.55 ± 0.25
1 M	6.1 ± 0.4	3.5 ± 0.5	-2.6 ± 0.4
Exp	6.3	1.7 ± 0.9	-2.5 ± 1.2

S9 Simulations at higher temperature

In this work, the simulations used in the fitting were performed at a temperature of 300 K, which is a value commonly used in molecular dynamics simulations. However, the experimental free energy differences used in the fitting refer to a temperature of 310 K. In order to quantify the dependence of the computed $\Delta\Delta Gs$ on temperature, we performed further control simulations for systems A1 and A2 at 310 K. Table S7 compares ΔGs computed at 300 K or 310 K, whereas Table S8 compares the $\Delta\Delta G$s. Differences are compatible with statistical error. These results suggest that the fitting is not affected by the choice of performing the simulations at 300 K rather than 310 K.

Table S7: ΔGs computed through alchemical computations and binless WHAM method, using the fit_AB parametrization. The rows correspond to different temperatures used in the simulations.

**T**	**A1 syn**	**A1 anti**	**A2 dup**	**A2 ss**
300 K	211.23 ± 0.18	217.27 ± 0.19	214.01 ± 0.35	210.40 ± 0.22
310 K	211.87 ± 0.18	217.65 ± 0.15	214.05 ± 0.24	210.87 ± 0.36

Table S8: $\Delta\Delta G$s computed through alchemical computations and binless WHAM method, using fit_AB parametrization. The first two rows correspond to different temperatures used in the simulation, and last row corresponds to the reference experimental values.

**T**	**A1**	**A2**
300 K	6.04 ± 0.26	3.6 ± 0.4
310 K	5.78 ± 0.23	3.18 ± 0.4
Exp	6.3	1.7 ± 0.9

It is also possible to extrapolate experimental and computational ΔGs from 300 K to 310 K by making use of following thermodynamics relationship:

$$\Delta G_{310} = \Delta G_{300} - (310 - 300)\Delta S \quad (19)$$

which can be applied to the calculation of $\Delta\Delta G$s, resulting in:

$$\Delta\Delta G_{310} = \Delta\Delta G_{300} - (310 - 300)\Delta\Delta S \tag{20}$$

or

$$\Delta\Delta\Delta G = -\Delta T \Delta\Delta S \tag{21}$$

By making use of this relation, we can investigate how $\Delta\Delta G$ would be affected for a change in temperature of 10 K, both for experimental and computational values. As far as the exerimental values are concerned, we compute $\Delta T \Delta\Delta S$ for systems B1-B5 by taking the difference between the ΔS measured for the methyated systems[14] and those for the unmethylated systems[15].[16] Results are reported in Table S9. Since we didn't find the experimental errors for the unmethylated systems, we assumed them to be identical to those obtained in the methylated systems. The changes in $\Delta\Delta G$ are small and dominated by their experimental error.

Table S9: $\Delta\Delta\Delta G$s for ΔT=10 K computed from experimental entropies

	**B1**	**B2**	**B3**	**B4**	**B5**
$-\Delta T \Delta\Delta S$ (kJ/mol)	0.6 ± 0.9	-1.0 ± 0.5	-0.5 ± 0.4	0.33 ± 0.20	0.3 ± 0.5

We then computed ΔSs from our simulations by making use of the relationship:

$$\Delta S = -\frac{\Delta G - \Delta U}{T} = -\frac{\Delta G - (\langle U \rangle_{\lambda=1} - \langle U \rangle_{\lambda=0})}{300} \tag{22}$$

$\Delta\Delta\Delta G$s for ΔT=10 K are shown in Table S10. Statistical errors were computed with blocked bootstrap. Also in this case, changes in $\Delta\Delta G$ are small and dominated by their statistical errors. A recalculation of the χ^2 using $\Delta\Delta G$ extrapolated at 310K returns a value χ^2 = 6.03, which is equivalent to the value reported in Fig. 4d using 300K results (χ^2 = 5.71), confirming that changing temperature does not affect the comparison between simulation and experiment.

Table S10: $\Delta\Delta\Delta G$s for ΔT=10 K estimated from computations

	**A1**	**A2**	**A3**	**A4**	**A5**
$-\Delta T \Delta\Delta S$ (kJ/mol)	0.1 ± 1.3	-0.57 ± 0.33	0.37 ± 0.36	-0.19 ± 0.36	-0.36 ± 0.22
	**B1**	**B2**	**B3**	**B4**	**B5**
$-\Delta T \Delta\Delta S$ (kJ/mol)	-0.75 ± 0.33	0.5 ± 0.4	-0.1 ± 0.6	0.0 ± 0.4	0.38 ± 0.39

S10 List of simulations

We here report the list of simulations performed in this work:

- For the 22 systems reported in Table S3, alchemical simulations were performed using Aduri, fit_A and fit_AB force-field parameters.
- For the 7 systems reported in Table S5, control alchemical simulations were performed at a higher salt concentration.
- For the 4 systems in Table S7, control alchemical simulations were performed at a higher temperature

This resulted in a total of $22 \times 3 + 7 + 4 = 77$ simulations. Each simulation was run with 16 replicas for 10 ns per replicas, for a total simulated time of $77 \times 16 \times 10\text{ns} = 12.32\mu\text{s}$.

The size of the simulated systems depended on the number of simulated nucleotides. For the smallest A1 system (one nucleoside), the setup included ≈ 1500 water molecules, 3 Na^+ and 3 Cl^- ions. Double stranded RNAs were simulated in boxes typically containing ≈ 6000 water molecules, the largest system being B4 with 7082 water molecules, 32 Na^+ and 14 Cl^- ions. Single stranded RNAs were simulated using slightly smaller boxes typically containing ≈ 4500 water molecules. The smallest systems were A4 and A5, which were solvated in less than 3000 water molecules.

S11 Structural snapshots

Figures from S9 to S18 shows snapshots for all the systems considered in this work. Snapshots were taken from simulations run with fit_AB parametrization and λ=1. The m6A hybrid nucleotides are shown with a broader and shiner pattern compared to other nucleotides.

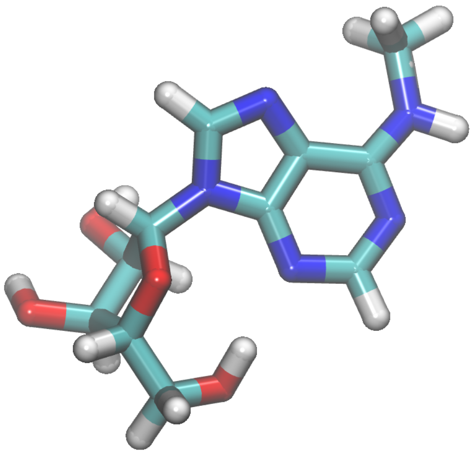

(a) *anti*

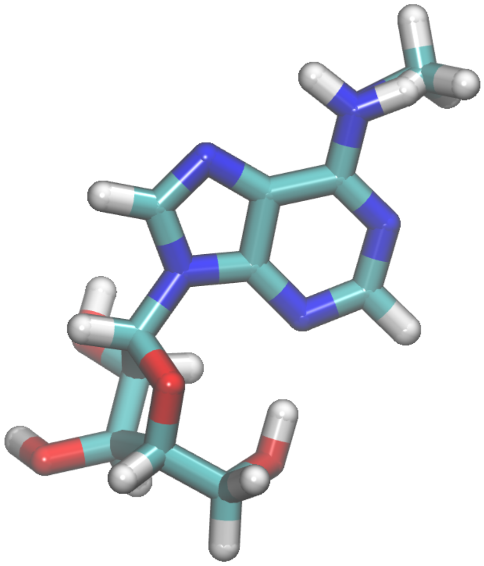

(b) *syn*

Figure S9: snapshots for A1 system

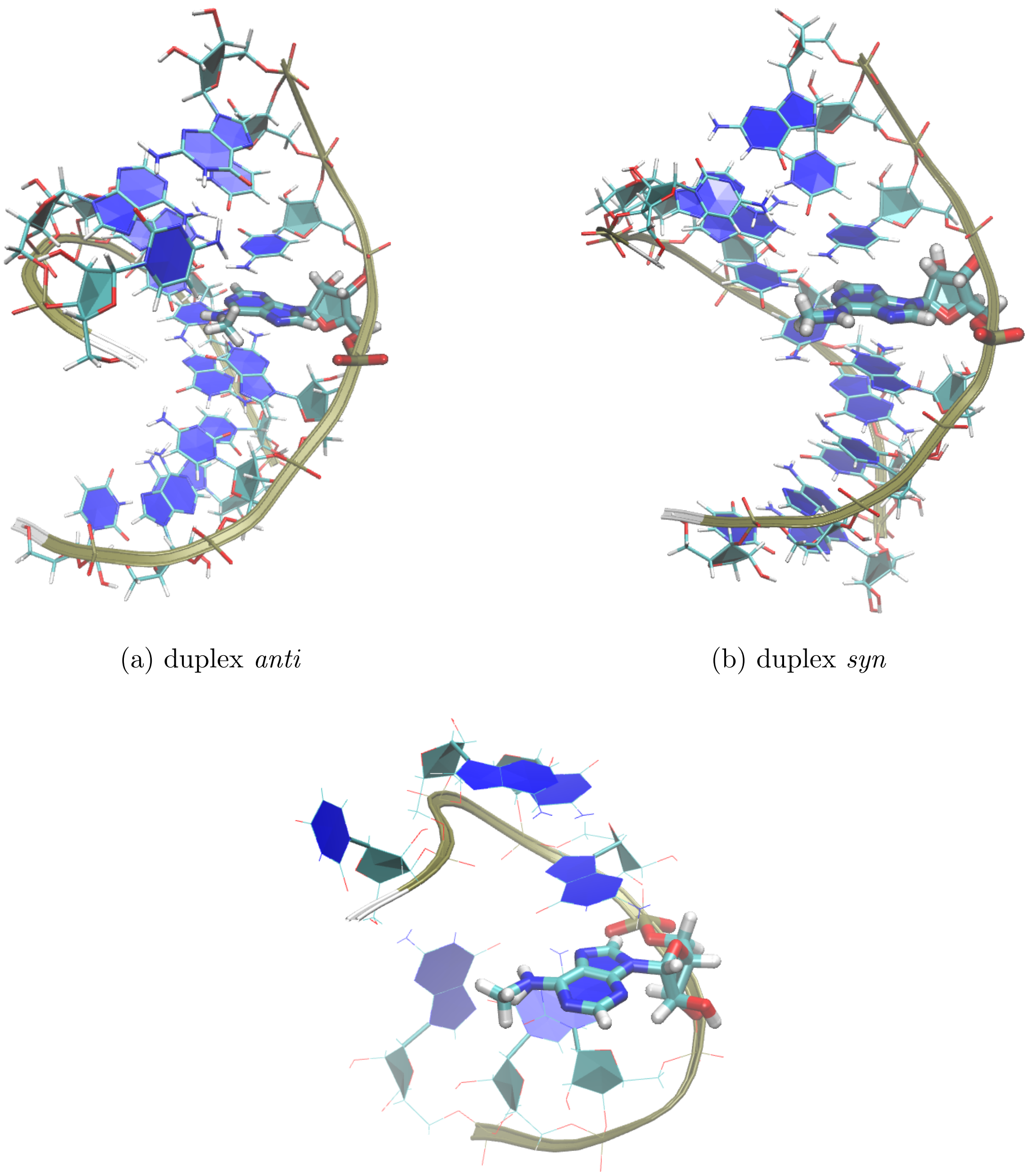

(a) duplex *anti*

(b) duplex *syn*

(c) single strand

Figure S10: snapshots for A2 system

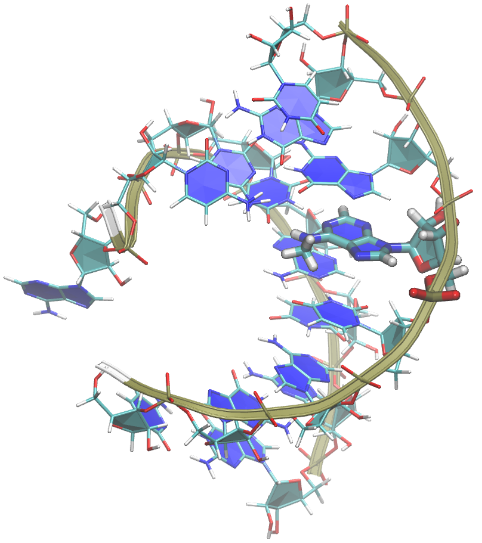

(a) duplex *anti*

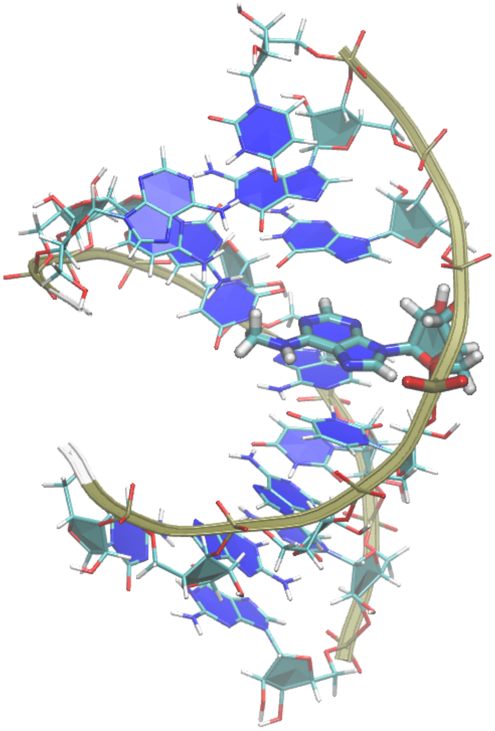

(b) duplex *syn*

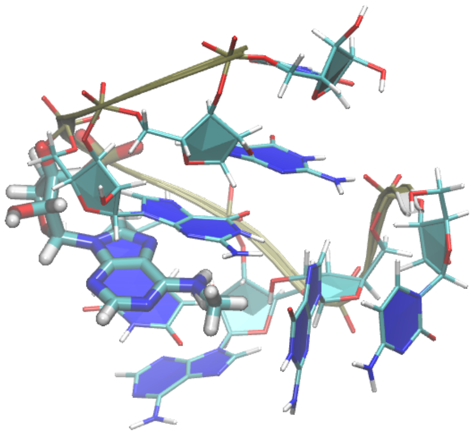

(c) single strand

Figure S11: snapshots for A3 system

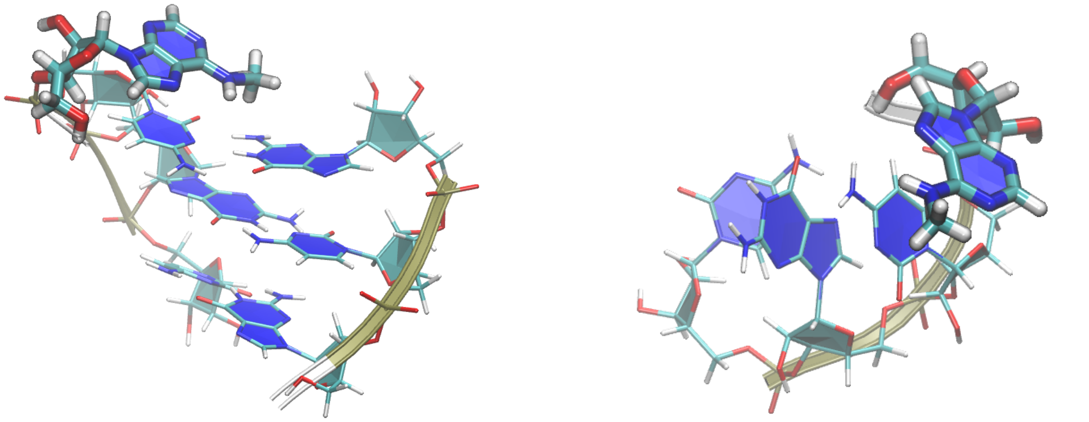

(a) duplex (b) single strand

Figure S12: snapshots for A4 system

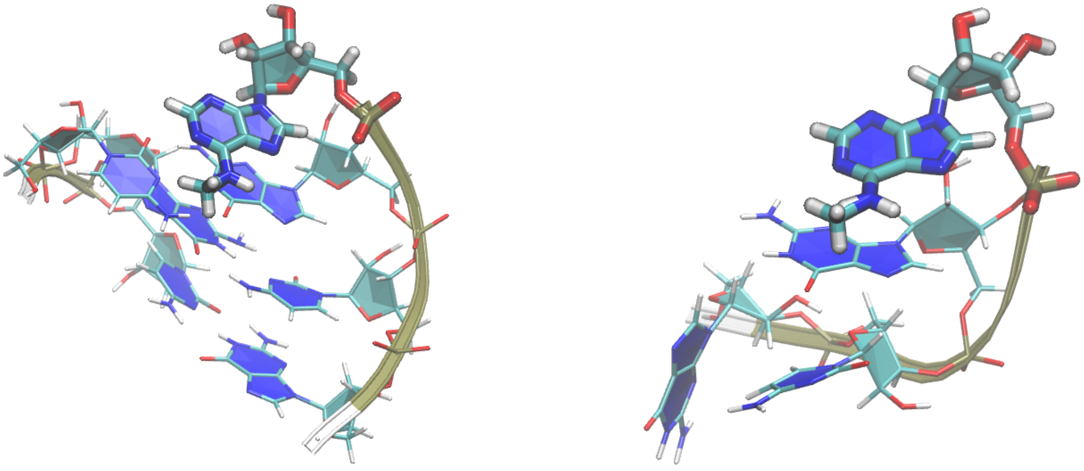

(a) duplex (b) single strand

Figure S13: snapshots for A5 system

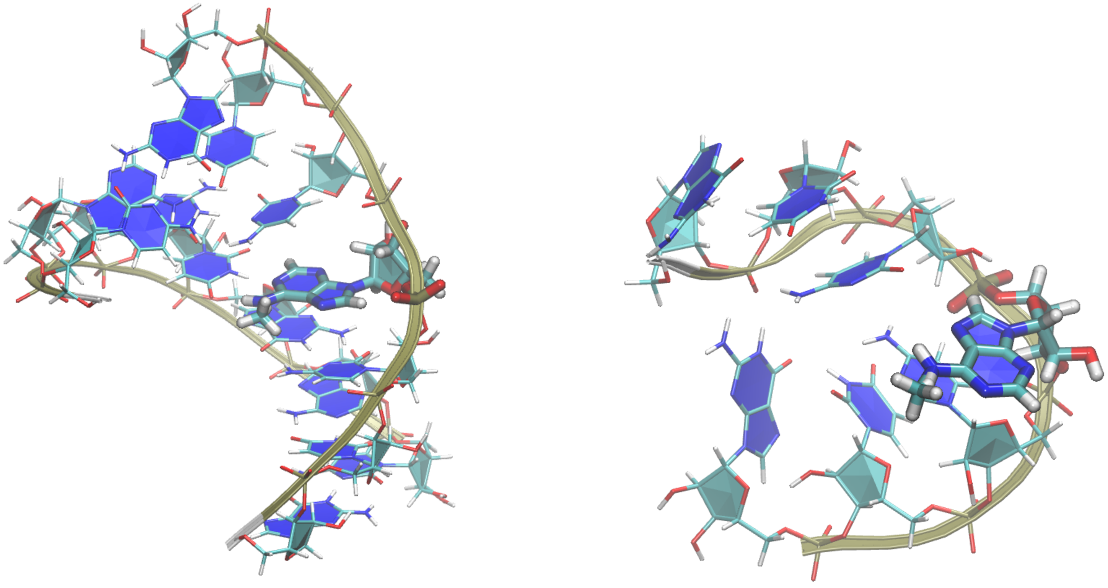

(a) duplex (b) single strand

Figure S14: snapshots for B1 system

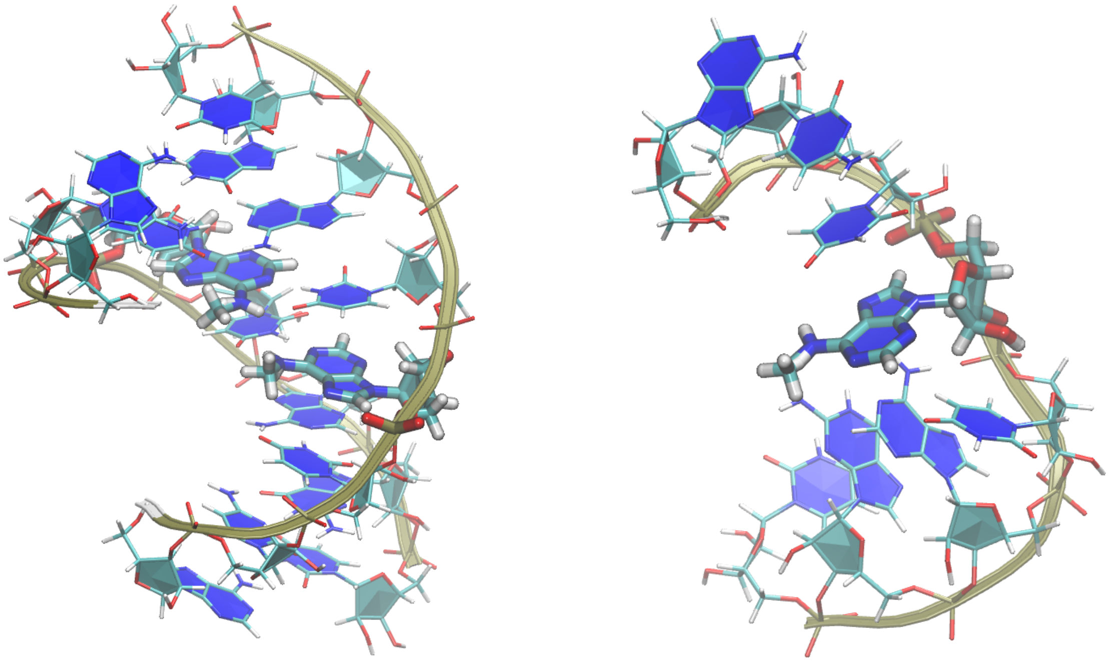

(a) duplex (b) single strand

Figure S15: snapshots for B2 system

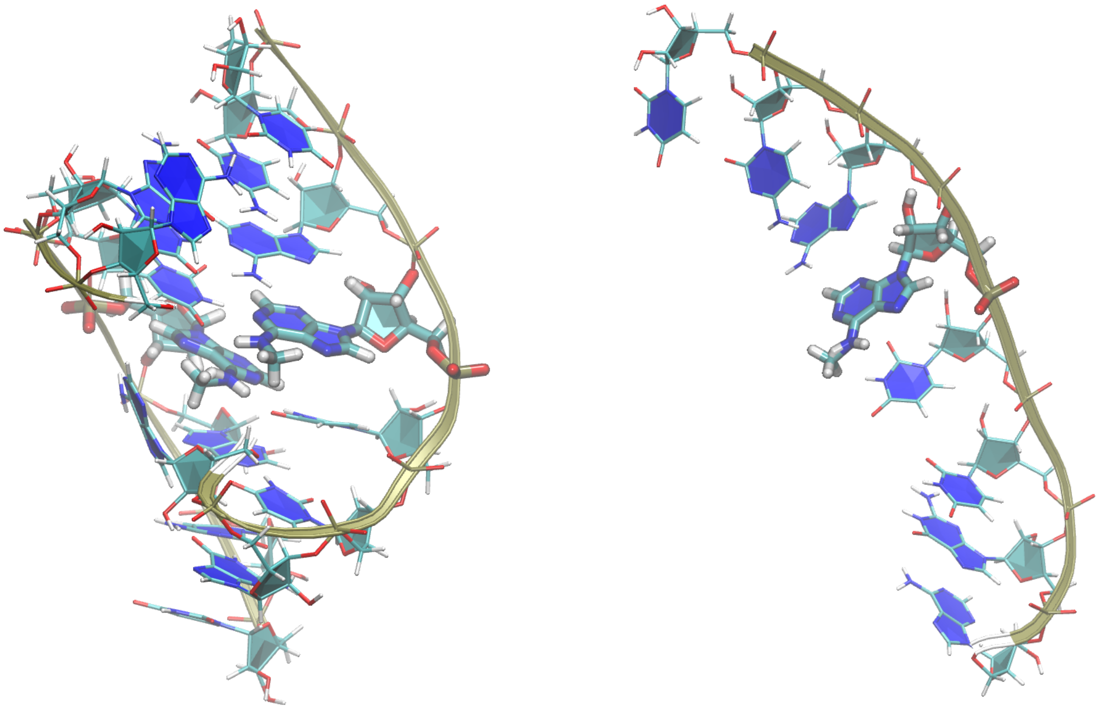

(a) duplex (b) single strand

Figure S16: snapshots for B3 system

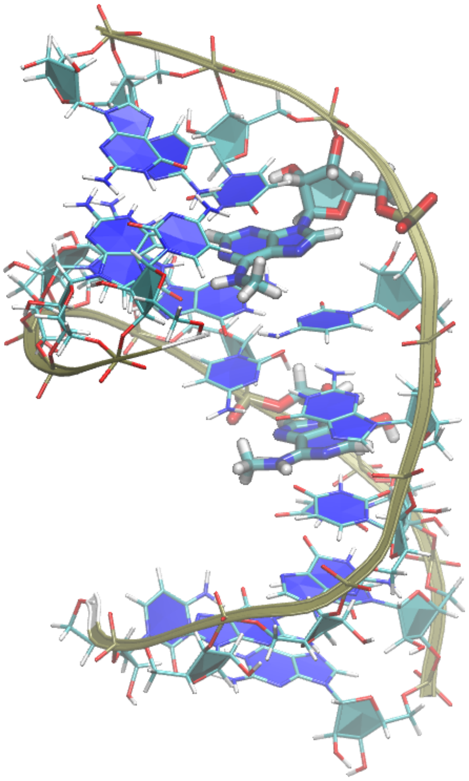

(a) duplex

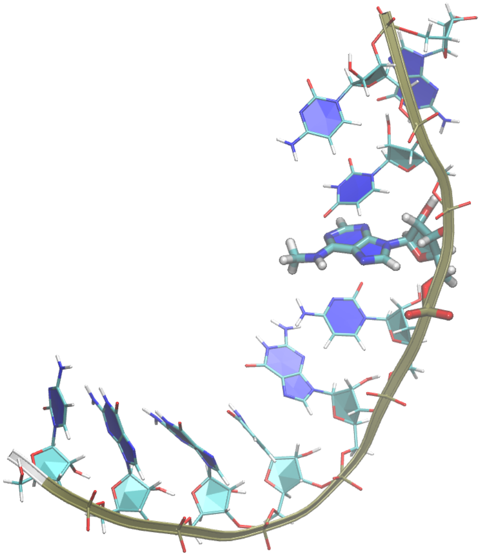

(b) single strand

Figure S17: snapshots for B4 system

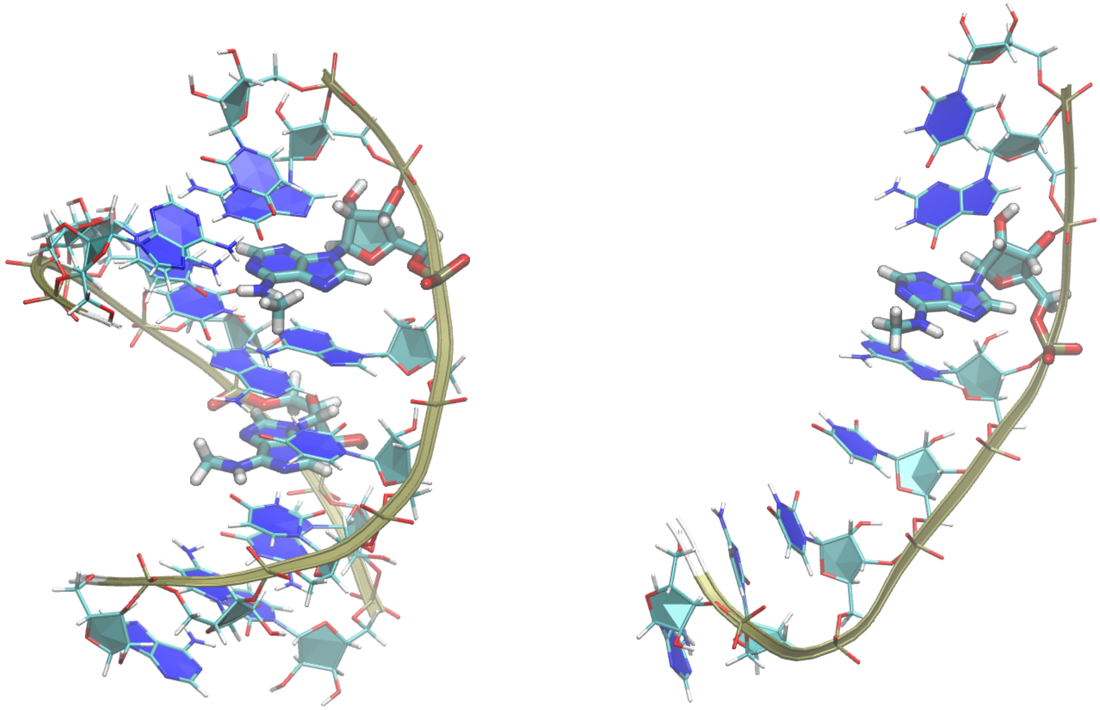

(a) duplex (b) single strand

Figure S18: snapshots for B5 system

References

(1) Aduri, R.; Psciuk, B. T.; Saro, P.; Taniga, H.; Schlegel, H. B.; SantaLucia, J. AMBER force field parameters for the naturally occurring modified nucleosides in RNA. *J. Chem. Theory Comput.* **2007**, *3*, 1464–1475.

(2) Cornell, W. D.; Cieplak, P.; Bayly, C. I.; Gould, I. R.; Merz, K. M.; Ferguson, D. M.; Spellmeyer, D. C.; Fox, T.; Caldwell, J. W.; Kollman, P. A. A second generation force field for the simulation of proteins, nucleic acids, and organic molecules. *J. Am. Chem. Soc.* **1995**, *117*, 5179–5197.

(3) Pérez, A.; Marchán, I.; Svozil, D.; Šponer, J.; Cheatham III, T. E.; Laughton, C. A.; Orozco, M. Refinement of the AMBER force field for nucleic acids: improving the description of α/γ conformers. *Biophys. J.* **2007**, *92*, 3817–3829.

(4) Zgarbová, M.; Otyepka, M.; Šponer, J.; Mládek, A.; Banáš, P.; Cheatham, T. E.; Jurečka, P. Refinement of the Cornell et al. nucleic acids force field based on reference quantum chemical calculations of glycosidic torsion profiles. *J. Chem. Theory Comput.* **2011**, *7*, 2886–2902.

(5) Bussi, G.; Laio, A. Using metadynamics to explore complex free-energy landscapes. *Nature Rev. Phys.* **2020**, *2*, 200–212.

(6) Tribello, G. A.; Bonomi, M.; Branduardi, D.; Camilloni, C.; Bussi, G. PLUMED 2: New feathers for an old bird. *Comput. Phys. Commun.* **2014**, *185*, 604–613.

(7) Souaille, M.; Roux, B. Extension to the weighted histogram analysis method: combining umbrella sampling with free energy calculations. *Comput. Phys. Commun.* **2001**, *135*, 40–57.

(8) Shirts, M. R.; Chodera, J. D. Statistically optimal analysis of samples from multiple equilibrium states. *J. Chem. Phys.* **2008**, *129*, 124105.

(9) Tan, Z.; Gallicchio, E.; Lapelosa, M.; Levy, R. M. Theory of binless multi-state free energy estimation with applications to protein-ligand binding. *J. Chem. Phys.* **2012**, *136*, 144102.

(10) Frisch, M.; et al., Gaussian 09, revision D01. *Gaussian Inc., Wallingford, CT* **2009**,

(11) Cieplak, P.; Cornell, W. D.; Bayly, C.; Kollman, P. A. Application of the multimolecule and multiconformational RESP methodology to biopolymers: Charge derivation for DNA, RNA, and proteins. *J. Comput. Chem.* **1995**, *16*, 1357–1377.

(12) Krepl, M.; Damberger, F. F.; von Schroetter, C.; Theler, D.; Pokorná, P.; Allain, F. H.-T.; Šponer, J. Recognition of N6-Methyladenosine by the YTHDC1 YTH Domain Studied by Molecular Dynamics and NMR Spectroscopy: The Role of Hydration. *J. Phys. Chem. B* **2021**, *125*, 7691–7705.

(13) Zhu, C.; Byrd, R. H.; Lu, P.; Nocedal, J. Algorithm 778: L-BFGS-B: Fortran subroutines for large-scale bound-constrained optimization. *ACM Trans. Math. Softw.* **1997**, *23*, 550–560.

(14) Kierzek, E.; Zhang, X.; Watson, R. M.; Kennedy, S. D.; Szabat, M.; Kierzek, R.; Mathews, D. H. Secondary structure prediction for RNA sequences including N6-methyladenosine. *Nat. Commun.* **2022**, *13*, 1–10.

(15) Xia, T.; SantaLucia, J.; Burkard, M. E.; Kierzek, R.; Schroeder, S. J.; Jiao, X.; Cox, C.; Turner, D. H. Thermodynamic Parameters for an Expanded Nearest-Neighbor Model for Formation of RNA Duplexes with Watson-Crick Base Pairs. *Biochemistry* **1998**, *37*, 14719–14735.

(16) Chen, J. L.; Dishler, A. L.; Kennedy, S. D.; Yildirim, I.; Liu, B.; Turner, D. H.; Serra, M. J. Testing the Nearest Neighbor Model for Canonical RNA Base Pairs: Revision of GU Parameters. *Biochemistry* **2012**, *51*, 3508–3522.
